# Brain energy metabolism in neurodegenerative disease

**DOI:** 10.1016/j.nbd.2026.107615

**Published:** 2026-11

**Authors:** Carmen Maria Lahr, Elle Wilson, Melanie Sengupta, Michela Bernini, Alexander Skupin

**Affiliations:** a Luxembourg Centre for Systems Biomedicine, University of Luxembourg, 6, Avenue du Swing, Esch-sur-Alzette, Luxembourg; b Department of Physics, University Luxembourg, 7, Avenue de la Faiencerie, Luxembourg, Luxembourg; c Department of Neurosciences, University of California San Diego, La Jolla, California, United States of America

**Keywords:** Brain energy metabolism, Parkinson's disease, Alzheimer's disease, Mitochondrial dysfunction

## Abstract

The brain is a highly complex organ composed of diverse, specialized cell types whose coordinated functions underpin cognition and behavior. While extensive research has elucidated neuronal signaling mechanisms, the metabolic interactions between distinct brain cells that sustain energy homeostasis are not yet comprehensively understood. Recently, brain energy metabolism has gained significant attention as a unifying factor in the pathogenesis of neurodegenerative diseases such as Alzheimer's and Parkinson's, potentially opening new avenues for therapeutic development. In this review, we provide an overview of brain energy metabolism, examine its role in neurodegeneration, and highlight current challenges and future perspectives in the field.

## 1 Introduction

The brain orchestrates a wide range of physiological functions, including sensation, motor control, cognition, and consciousness. Despite representing only 2% of total body mass, the brain consumes around 20% of the body's total energy supply (Padamsey and Rochefort, 2023). Around 80% of this energy is expended by neurons to maintain ion gradients, regulate neurotransmitter turnover, and facilitate synaptic transmission (Beard et al., 2022; Yu et al., 2018; Hyder et al., 2013; Watts et al., 2018; Attwell and Laughlin, 2001). Most of this energy demand is met through glucose metabolism, which fuels mitochondrial oxidative phosphorylation (OXPHOS) to generate adenosine triphosphate (ATP). Neuronal electrical activity, neurotransmission, and cellular homeostasis are all critically dependent on this ATP supply.

Because the brain operates so close to its energetic limits, it becomes particularly vulnerable to disruptions. Even modest reductions in energy supply or impairments in metabolic coordination can compromise neuronal function, destabilize networks, and create conditions that favor cellular stress. These vulnerabilities become increasingly evident during aging, when cerebral glucose metabolism progressively declines in regions such as the frontal, temporal, and parietal lobes (Pak et al., 2023). This reduction in energy availability lowers neuronal resilience and creates a permissive environment for neurodegenerative processes (Pak et al., 2023). In Alzheimer's disease (AD) and Parkinson's disease (PD), this bioenergetic fragility is compounded by mitochondrial dysfunction, impaired substrate utilization, and disrupted neuron–glia metabolic coupling (Cunnane et al., 2020). Such defects in energy production and utilization are now recognized as early and potentially causal contributors to neurodegeneration, rather than simply downstream consequences of neuronal loss (Yin et al., 2016). The growing recognition of energy metabolism as a key determinant of brain health has spurred the development of brain energy rescue strategies aimed at restoring or stabilizing metabolic function in aging and neurodegenerative disease (Cunnane et al., 2020). However, to design effective interventions, it is essential to understand how energy is produced, distributed, and utilized within the brain at both the cellular and molecular levels, and how these processes are differentially affected in health, aging, and disease.

Whereas previous reviews have examined brain metabolism from complementary perspectives, including systemic metabolic alterations in aging and neurodegeneration (Camandola and Mattson, 2017), the astrocyte–neuron lactate shuttle and lactate signaling (Magistretti and Allaman, 2015, Magistretti and Allaman, 2018), and the broader integration of glucose metabolism with brain function (Dienel, 2019), this review organizes brain bioenergetics around the division of metabolic functions among the major brain cell populations, including neurons, astrocytes, oligodendrocytes, and microglia. Rather than focusing on individual metabolic pathways or specific metabolic hypotheses, we examine how cell-type–specific metabolic specialization, mitochondrial function, and intercellular metabolic coupling collectively shape neuronal vulnerability in neurodegenerative disease. Using AD and PD as principal disease models, we further integrate recent advances in mitochondrial dynamics, glial immunometabolism, lipid droplet biology, patient-derived cellular models, metabolic imaging biomarkers, and emerging metabolic therapies. By linking cellular energy demands to disease-specific vulnerabilities, this review provides an updated framework that connects brain metabolism across molecular, cellular, and systems levels.

## 2 Principles of brain energy organization

Glucose as the primary energy source supports distinct metabolic pathways within the brain, each tailored to different energetic demands. Although most ATP ultimately arises from mitochondrial OXPHOS, a smaller portion is produced through glycolysis in the cytoplasm, together accounting for roughly 95% of ATP generated from glucose catabolism (Wilson, 2017). These pathways serve complementary roles: glycolysis provides rapid, activity-dependent energy at synapses, whereas mitochondria supply a steadier output that sustains broader cellular processes (Ashrafi et al., 2017).

Glucose uptake into brain cells is mediated by a family of facilitative transport proteins known as glucose transporters (GLUTs) (Stringer et al., 2015). At the blood–brain barrier (BBB), glucose is transported from the circulation into the brain's interstitial fluid primarily via GLUT1 expressed on endothelial cells (Fig. 1; Holman, 2020). From there, neurons predominantly take up glucose via GLUT3, while astrocytes predominantly utilize GLUT1. GLUT2 has additionally been reported in specific astrocyte populations and may contribute to glucose sensing (Marty et al., 2005; Koepsell, 2020).

**Fig. 1:**
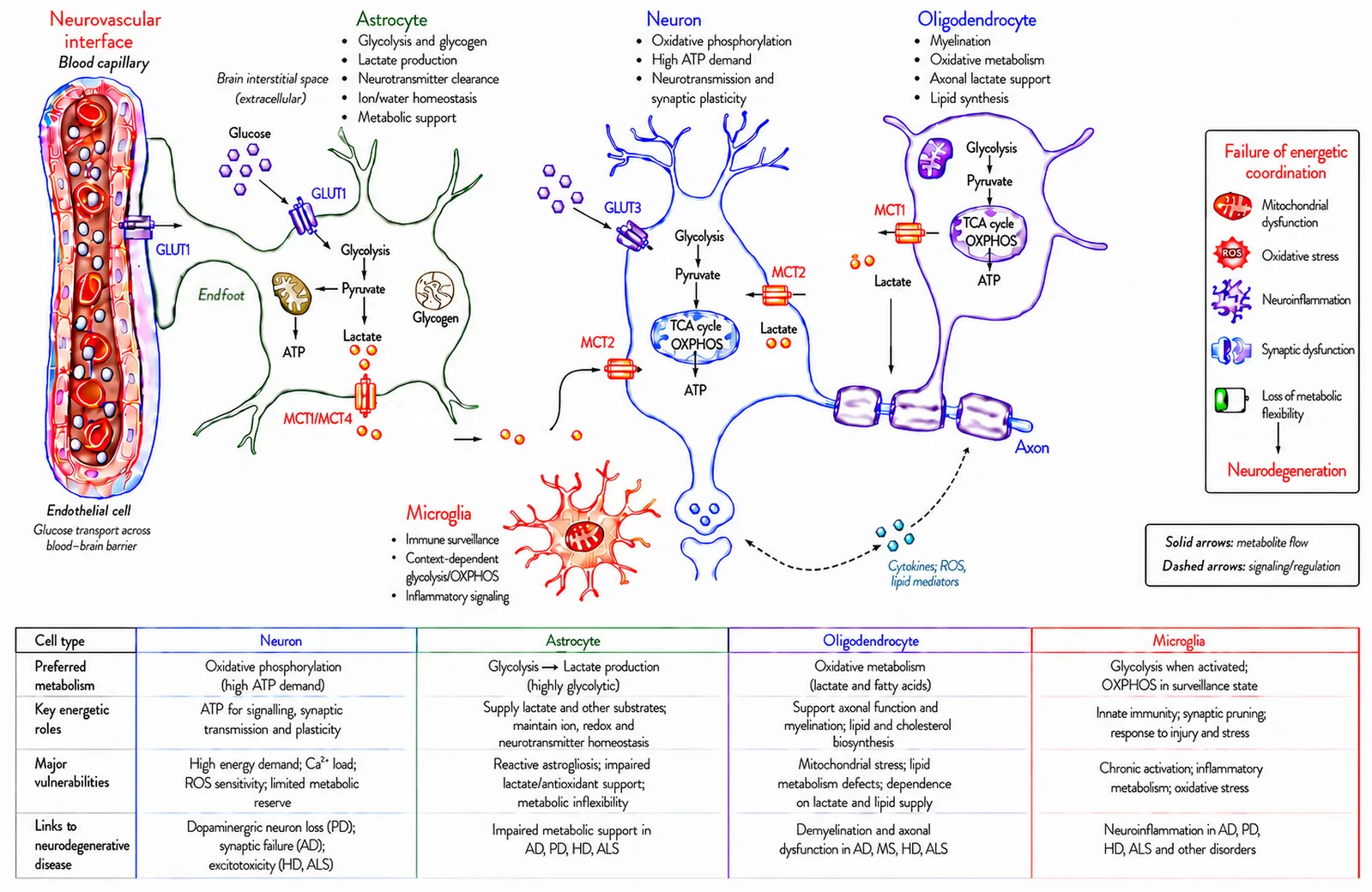
Cell-type contribution to brain energy metabolism. Brain energy metabolism relies on coordinated interactions among neurons, astrocytes, oligodendrocytes, microglia, and the neurovascular unit. Neurons consume the majority of ATP to sustain electrical activity, synaptic transmission, ion homeostasis, and intracellular transport. Astrocytes regulate glucose uptake, glycogen storage, lactate production, neurotransmitter recycling, neurovascular coupling, and lipid metabolism, thereby providing metabolic support to neurons. Oligodendrocytes contribute to axonal energy homeostasis through myelination and the transfer of metabolic substrates such as lactate and TCA-cycle intermediates. Microglia influence brain metabolism through immunometabolic reprogramming, inflammatory signaling, and the clearance of cellular debris. Together, these cell types form an integrated metabolic network that maintains brain energy homeostasis and whose disruption contributes to neurodegeneration.

This tightly regulated transport ensures a continuous glucose supply despite fluctuations in peripheral glucose levels, maintaining cerebral glucose concentrations within a relatively stable range (Koekkoek et al., 2017). Once inside the cytosol, glucose is phosphorylated to glucose-6-phosphate and enters the glycolytic pathway (Verberne et al., 2014). The resulting pyruvate can either be reduced to lactate by the lactate dehydrogenase (LDH) or transported into mitochondria, where it enters the tricarboxylic acid (TCA) cycle and fuels OXPHOS via the electron transport chain (ETC) (Fig. 2). Alternatively, the glycolytic intermediate glucose-6-phosphate can be diverted into the pentose phosphate pathway (PPP). Unlike glycolysis, which is mainly catabolic, the PPP supports anabolic and protective functions by generating NADPH required for lipid and steroid synthesis, cytochrome P450 activity, and the regeneration of glutathione, a key antioxidant involved in detoxifying reactive oxygen species (ROS) (Rassow et al., 2008). These pathways provide the majority of cellular ATP and are therefore critical for maintaining neuronal function and survival.

**Fig. 2:**
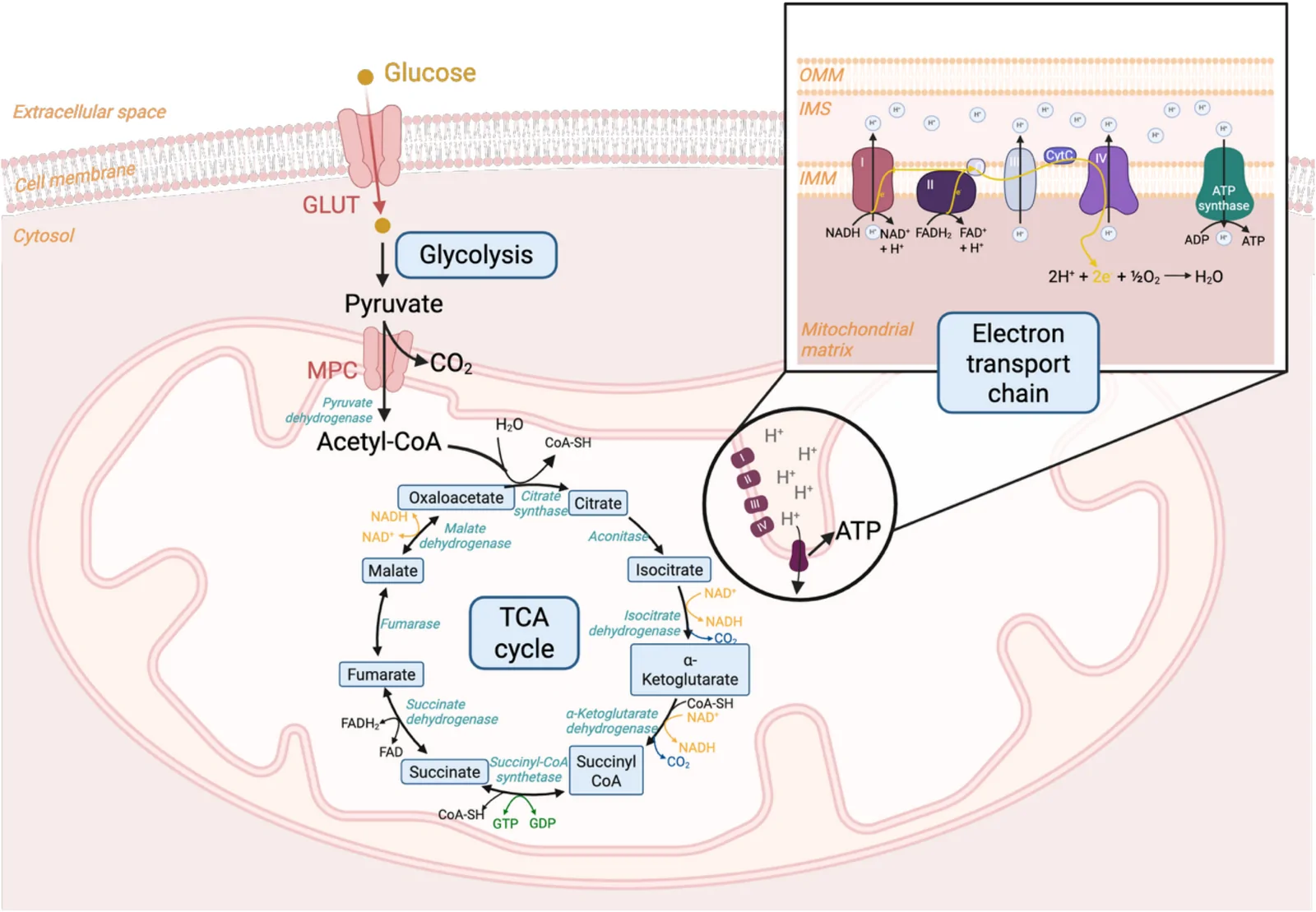
Glucose metabolism in the cell. Following glycolysis, pyruvate enters the mitochondrion and is transferred into acetyl-CoA which enters the TCA cycle which is located to the mitochondrial matrix. Several side products of the TCA cycle, including NADH and FADH_2_ will be used in the ETC as electron donors. Proteins of the ETC (complex I – IV) are located at the inner mitochondrial membrane (IMM) and transport protons (H^+^) to the intermembrane space between IMM and outer mitochondrial membrane (OMM). Complex I and II additionally accept electrons from the oxidation of NADH and FADH_2_, which are passed to coenzyme Q, complex III, cytochrome C and finally complex IV, where they are transferred to the condensation reaction which generates water. The proton gradient, generated by complex I – IV, generates a membrane potential which drives the ATP synthase to produce ATP. Created with BioRender.

Cellular glucose handling is dynamically linked to neurovascular coupling, a process whereby increased neuronal activity triggers vasodilation, enhancing cerebral blood flow and glucose delivery (Sundqvist et al., 2022). Components of the neurovascular unit including endothelial cells, pericytes, astrocytic endfeet, and neurons integrate metabolic signals with vascular responses to match substrate availability to regional energy demands. This tight coordination between blood flow, oxygen consumption, and glucose utilization forms the physiological basis of functional neuroimaging techniques such as magnetic resonance imaging (Yang et al., 2023; Xu et al., 2023).

While glucose metabolism supports most cerebral ATP production, the brain also relies on additional substrates that contribute under specific physiological conditions. These alternative fuels differ in availability, regulation, and functional role, forming a spectrum that ranges from rapidly mobilizable local stores to systemically driven energy supplies.

The most rapidly accessible of these auxiliary substrates is glycogen, which provides a short-term buffer when energy demand rises, or glucose supply momentarily drops. Although total glycogen stores in the brain are low, they represent the brain's energy reserve (Brown and Ransom, 2007). This reserve is localized primarily in astrocytes (Calì et al., 2019), as demonstrated through electron microscopy and the astrocyte-specific distribution of glycogen phosphorylase (Phelps, 1972; Pfeiffer-Guglielmi et al., 2003). In the glycogen shunt, glucose is transiently stored as glycogen via glycogen synthase (Staricha et al., 2020). When energy demands increase, this glycogen can be mobilized and broken down into glucose-6-phosphate by glycogen phosphorylase, which then feeds into glycolysis (Bak et al., 2018). Under physiological conditions, glycogen synthesis in neurons is actively suppressed (Vilchez et al., 2007) and a failure of this suppression can induce neurodegenerative diseases characterized by polyglucosan accumulation (Cavanagh, 1999). Under hypoglycemic conditions, astrocytes specifically metabolize glycogen before transferring the resulting lactate to adjacent neurons (Staricha et al., 2020). Glycogen accumulation can be observed at times of decreased neuronal activity, e.g., with the use of anesthetics (Phelps, 1972), while neuronal stimulation leads to transient glycogen depletion in nearby astrocytes, further indicating its energy buffer function (Staricha et al., 2020). Furthermore, failure to effectively break down glycogen is observed in multiple neurodegenerative disorders (Bak et al., 2018).

As glycogen is broken down in astrocytes, lactate is generated and can be shuttled to neighboring neurons, providing a rapidly available, activity-responsive fuel. Several studies indicate that under certain conditions, neurons preferentially use lactate as an energy substrate (Xue et al., 2022). For example, glucose uptake decreases in primary cultured cortical neurons exposed to glutamate (Porras et al., 2004), while PET imaging and [18F]-fluoro-deoxy-glucose tracing show increased lactate uptake and reduced glucose metabolism following intense exercise (Attwell and Laughlin, 2001; Kemppainen et al., 2005). Similarly, stable isotope tracing experiments indicated that up to 75% of oxidative metabolism in neurons can be supported by lactate (Bouzier-Sore et al., 2006). These findings contributed to the development of the astrocyte–neuron lactate shuttle (ANLS), an influential model of neuron-glia metabolic coupling in which astrocyte-derived lactate supports neuronal metabolism (Pellerin and Magistretti, 1994).

In contrast to glycogen and lactate, which are tightly linked to local brain activity, ketone bodies serve as a major energy source when systemic metabolic conditions shift, such as during fasting, prolonged exercise, ketogenic diets, or early development (Omori et al., 2022; Vijay et al., 2015). Importantly, ketone uptake across the BBB depends on circulating ketone levels rather than neuronal activity (Cunnane et al., 2016). Among circulating ketone bodies, D-β-hydroxybutyrate (BHB) is the predominant substrate used by the brain during fasting or carbohydrate restriction. Once in the brain, BHB is converted to acetoacetate then to acetoacetyl-CoA (Jensen et al., 2020). Acetoacetyl-CoA yields two acetyl-CoA molecules, which enter the TCA cycle and generate ATP (Pietrocola et al., 2015). Fatty acids can also contribute to TCA-cycle intermediates, and astrocytes—rather than neurons—are the primary site of mitochondrial fatty acid oxidation in the brain (Zhang et al., 2023). Beyond fasting, ketones are also essential during development: the neonatal brain, derives nearly half of its energy from ketone bodies, and its rate of ketone extraction is up to 40-fold higher than in adults (Cotter et al., 2011). Additionally, ketones provide the majority of the carbon needed to synthesize essential brain lipids in infants, such as saturated and monosaturated fatty acids and cholesterol (Cunnane, 2018).

Given these major energy producing pathways, it is important to understand how this energy is allocated during brain activity. Classic estimates indicate that neurons draw the majority of the metabolic supply (up to 80%), with glial cells accounting for the remainder through their support roles (Attwell and Laughlin, 2001). A large portion of this energy is utilized in the generation and conduction of action potentials, where restoration of ion gradients by the Na^+^/K^+^-ATPase constitutes a significant proportion of energy expenditure associated with neuronal signaling (Meyer et al., 2022). Beyond action potential propagation, synaptic transmission imposes additional metabolic demands, including the maintenance of resting membrane potential, synaptic vesicle homeostasis (filling, transport and recycling), and neurotransmitter turnover. These processes further rely on ATP-dependent mechanisms such as Ca^2+^ clearance by plasma-membrane Ca^2+^-ATPase (PMCA) and presynaptic ion channel activity, contributing significantly to the overall energetic burden of synaptic function (Faria-Pereira and Morais, 2022).

## 3 Mitochondria as integrators of neuronal vulnerability

Given the specifically high energy demand in the brain, neuronal function relies heavily on efficient mitochondrial ATP production (Murali Mahadevan et al., 2021) and, unsurprisingly, mitochondrial dysfunction represents a common hallmark across numerous neurodegenerative disorders (Mlody et al., 2016; Arena et al., 2022; Murali Mahadevan et al., 2021; Cunnane et al., 2020).

A well-studied example of impaired mitochondrial function is complex I deficiency. Complex I of the ETC oxidizes NADH + H^+^ to NAD^+^, while simultaneously pumping protons into the intermembrane space, thereby contributing to the proton motive force required for ATP synthesis. In vitro studies showed that deletion of the complex I subunit *Ndufs4* abolishes complex I activity in mesencephalic neurons (Choi et al., 2011). Interestingly, however, dopaminergic *Ndufs4*−/− neurons retain viability despite this loss of function, suggesting unique metabolic adaptations within this neuronal subtype. Complex I deficiency is also a well-established hallmark of PD (Murali Mahadevan et al., 2021; Walter et al., 2019) as detailed below. Recent findings also underline that mitochondrial metabolic capacity influences the speed of neuronal development in a species-specific manner. Pharmacological enhancers of mitochondrial metabolism, such as AlbuMAX-GSK, were shown to accelerate neuronal differentiation (Iwata et al., 2023), underscoring the pivotal role of mitochondrial function not only in mature neurons but also during neurodevelopment.

Apart from energy metabolism, mitochondria perform numerous essential cellular functions, including biosynthesis of amino acids, fatty acids, NADH, and ketone bodies, regulation of apoptosis by BCL-2 macropores and cytochrome C release (Murali Mahadevan et al., 2021). They are also a major source of ROS which causes oxidative damage on cells and cell structures and contributes to aging and neurodegeneration, e.g., by activating the inflammasome (Borsche et al., 2021; Haque et al., 2020). Furthermore, mitochondria are important Ca^2+^ storage compartments where Ca^2+^ drives TCA cycle activity through concentration dependent activity of the TCA enzymes isocitrate and α-ketoglutarate dehydrogenases and the pyruvate dehydrogenase (Voorsluijs and Skupin, 2025).

Mitochondrial structure and membrane organization largely contribute to their function. The highly folded inner membrane forms cristae that increase surface area and create a compartmentalization to maintain ion gradients, such as the proton gradient that drives OXPHOS. The membrane architecture also allows for the lineup of numerous transmembrane complexes, including the ETC subunits. Disrupted or misshaped mitochondria are observed in a range of diseases, including PD, where altered morphology often reflects underlying defects in membrane organization, fusion-fission dynamics, or bioenergetics. Studies in cells derived from patients with the neurodegenerative Leigh syndrome demonstrate that disrupted mitochondrial ultrastructure is closely linked to disease progression (Siegmund et al., 2018). Using in situ cryo-electron tomography, Siegmund et al. reported pronounced abnormalities in crista membrane organization in patient fibroblasts, including increased crista width and curvature, which may impair ATP synthase dimerization and consequently compromise oxidative phosphorylation (Cogliati et al., 2013; Strauss et al., 2008; Garcia et al., 2019; Siegmund et al., 2018). The observed cristae abnormalities in Leigh syndrome suggest that similar ultrastructural defects may be a common feature in other neurodegenerative diseases.

Notably, mitochondrial morphology and motility are not static but vary substantially depending on a cell's differentiation state, metabolic requirements, and spatial context (Murali Mahadevan et al., 2021): For example, stem cells contain predominately round mitochondria with poorly developed cristae, whereas mature neurons display elongated and cristae-rich mitochondria (Mlody et al., 2016; Tsogtbaatar et al., 2020). While axonal mitochondria often appear as well-defined organelles, they typically form interconnected networks in other cellular regions through continuous fusion and fission. These dynamic morphological changes allow mitochondria to rapidly adapt to the cell's metabolic and energetic requirements. Disruption of this balance is associated with various disorders, including metabolic diseases and neurodegeneration (Bertholet et al., 2016; Qi et al., 2013). Excessive fission and fragmentation by the dynamin-1-related protein (Drp1), together with the adapter protein Fis1, leads to fragmentation and contributes to neuronal loss by promoting apoptosis, autophagy, and necrosis (Joshi et al., 2019; Qi et al., 2013). Mitochondrial fragmentation may also trigger inflammatory responses and neuronal death, as demonstrated by the microglial release of fragmented mitochondria (Joshi et al., 2019). However, the impact on mitochondrial dynamics remains controversial, as exemplified by the PD-associated LRRK2. Several studies report that pathogenic LRRK2 activity promotes Drp1-mediated mitochondrial fission, resulting in increased mitochondrial fragmentation and reduced elongation (Walter et al., 2019; Su and Qi, 2013). In contrast, other studies describe increased mitochondrial elongation and interconnectivity in LRRK2 G2019S models, which has been attributed to impaired mitochondrial fission (Mortiboys et al., 2010). Consistent with this, reduced levels of active, phosphorylated Drp1 have been reported in LRRK2 G2019S cells (Yue et al., 2015). These divergent findings have been summarized in recent reviews, highlighting impaired fission as a potential mechanism underlying elongated mitochondrial networks in LRRK2-associated PD (Wang et al., 2023; Boecker, 2023). Thus, LRRK2-associated PD exemplifies the broader uncertainty surrounding how pathogenic stressors modulate mitochondrial fission pathways, underscoring the need for context-dependent interpretation of mitochondrial morphology phenotypes. Using live-cell structured illumination microscopy, a recent study distinguished two mechanistically and functionally distinct types of mitochondrial fission. Only the peripheral fission was mediated by Fis1 and led to mitophagy (Kleele et al., 2021), whereas fission in the midzone resulted in mitochondrial biogenesis and proliferation. The small-molecule inhibitor P110 selectively blocks the Drp1/Fis1-mediated peripheral fission, thereby reducing the number of damaged extracellular mitochondria, improving macrophage function (Mukherjee et al., 2022), and enhancing dopaminergic neuron survival (Qi et al., 2013). In addition, P110 is therapeutically promising in Amyotrophic lateral sclerosis (ALS), where it has been reported to delay disease progression in mouse models (Dorn, 2023). Beyond genetic and signaling-driven regulation of fission, recent evidence suggests that mitochondrial dynamics are directly coupled to the cellular energy state. In this context, hexokinase-1, the first enzyme of glycolysis, has been identified as an endogeneous energy-stress sensor. Under conditions of low ATP availability, hexokinase-1 forms ring-like structures around mitochondria, which inhibits Drp1-mediated mitochondrial fission, thereby stabilizing mitochondrial networks during metabolic stress (Pilic et al., 2024).

Dysfunctional mitochondria are removed through mitophagy, a quality control process in which damaged mitochondria are selectively targeted for degradation. A key regulator of mitophagy is Miro, a mitochondrial membrane protein and transport adapter that connects mitochondria to motor proteins and the cytoskeleton to enable mitochondrial movement (Eberhardt et al., 2020; Hsieh et al., 2016). For mitophagy to proceed, Miro must be removed to halt mitochondrial movement and allow the isolation of damaged mitochondria (Hsieh et al., 2016). This clearance mechanism also limits accumulation of ROS generated by dysfunctional organelles (Von Stockum et al., 2016).

In parallel, the mitochondria-associated ER membrane (MAM) serves as a critical structural hub coordinating ER–mitochondria communication. MAMs regulate Ca^2+^ signaling, phospholipid transfer, and act as platforms for mitochondrial fission and fusion events (Liu and Zhu, 2017). In pathological contexts, MAM structure becomes disrupted—for example, MPTP-induced PD models show altered MAM morphology, indicating that ER–mitochondria coupling is highly sensitive to mitochondrial stress (Liu et al., 2025).

Together, these processes highlight how tightly mitochondrial morphology, function, and quality control are integrated, and why disturbances in any of these layers can have profound consequences for neuronal health.

## 4 Cell-type-specific metabolic specialization

Brain energy metabolism is organized in a cell-type–specific manner (Fig. 1), with neurons and astrocytes exhibiting distinct but complementary metabolic profiles (Allen and Barres, 2009). Astrocytes extend highly branched processes that closely associate with synapses and blood vessels, forming a structural and metabolic interface between neurons and the vasculature (Shigetomi et al., 2010). One model of metabolic support is the astrocyte–neuron lactate shuttle (ANLS) (Pellerin and Magistretti, 1994). According to this model, astrocytes preferentially utilize glycolysis, producing lactate that is released into the extracellular space and taken up by neurons via monocarboxylate transporters. Neurons convert the lactate into pyruvate which enters mitochondria and fuels the TCA cycle and OXPHOS (Bonvento and Bolaños, 2021; Mächler et al., 2016; Boumezbeur et al., 2010). Data on rat brains also suggest the astrocyte-neuron transport to be essential for memory formation (Suzuki et al., 2011).

Part of the rationale underlying the ANLS framework stems from intrinsic metabolic differences between the two cell types. Astrocytes express high levels of the glycolytic regulator phosphofructokinase (PFKFB3), whereas neurons maintain low levels of this enzyme due to higher turnover rates (Almeida et al., 2004). As a consequence, a greater proportion of glucose-6-phosphate in neurons can enter the PPP, rather than glycolysis. By prioritizing NADPH production over glycolytic ATP generation, neurons protect themselves against oxidative stress. Disruption of this balance, such as through overexpression of phosphofructokinase, leads to a shift in glucose-6-phosphate usage toward glycolysis, decrease in NADPH levels, and increase in ROS levels, finally inducing apoptosis (Bonvento and Bolaños, 2021; Herrero-Mendez et al., 2009). Impaired metabolic support by astrocytes is increasingly implicated in neurodegeneration (Schmidt et al., 2011), particularly in disorders characterized by mitochondrial dysfunction and disturbed energy homeostasis, such as PD (Dawson and Dawson, 2003; Mounsey and Teismann, 2010). While ANLS offers an influential model of glia–neuron metabolic coupling, extensive evidence indicates that its applicability may be context-dependent, and several studies have challenged the notion that lactate is the preferential neuronal fuel during activity (Hall et al., 2012; Díaz-García et al., 2017; Díaz-García and Yellen, 2018; Dienel, 2019). The review by Dienel (2019) provides an extensive critical evaluation of the ANLS hypothesis. A major source of controversy arises from studies employing genetically encoded metabolic biosensors, which enable real-time measurements of intracellular glucose, lactate, and ATP dynamics in defined cell populations. Using such approaches, Díaz-García et al. (2017) reported that neuronal stimulation rapidly increases neuronal glycolysis, indicating that neurons can directly increase glucose utilization during acute activity. These findings challenge the interpretation that activity-dependent ATP production relies primarily on astrocyte-derived lactate. In contrast, in vivo imaging and metabolic tracing studies have provided evidence consistent with astrocyte-to-neuron lactate transfer and with neuronal lactate utilization, supporting key aspects of the ANLS model (Mächler et al., 2016; Magistretti and Allaman, 2018). Methodological differences likely contribute to these divergent conclusions. Biosensor-based studies typically measure rapid, cell-specific metabolic responses during acute stimulation, whereas isotope-tracing and in vivo imaging approaches assess substrate fluxes over different spatial and temporal scales. Furthermore, substrate preference may depend on developmental stage, neuronal subtype, brain region, activity level, and disease state. Rather than supporting a universal metabolic strategy, current evidence suggests that neurons can utilize both glucose and lactate, with the relative contribution of each substrate varying according to physiological and pathological context (Dienel, 2019; Díaz-García and Yellen, 2018). Consequently, neuron–glia metabolic coupling is increasingly viewed as a flexible and adaptive system rather than a single, invariant pathway.

### 4.1 Neurons

Neuronal energy metabolism is tightly linked to electrical activity (Sharp et al., 1975) and energy consumption can reach levels similar to that in muscle cells (Ames, 2000; Brown and Ransom, 2007). Even at rest, neurons consume large amounts of ATP to maintain ion gradients, fuel vesicle cycling, and sustain axonal and dendritic transport (Lennie, 2003).

A defining feature of neuronal metabolism is its spatial compartmentalization. Owing to their small soma and long processes, neurons must control their energy supply locally. One key mechanism is the controlled transport and distribution of mitochondria, enabling their enrichment at sites of high metabolic demand, such as synapses (López-Doménech and Kittler, 2023). For example, early ultrastructural studies in macaques revealed neuronal mitochondria to be concentrated in glutamatergic synapses and unmyelinated axons, consistent with the elevated energetic requirements (Wong-Riley, 1989). By dynamically positioning mitochondria at sites of highest ATP demands, neurons maintain a tight coupling between activity and energy supply that is essential for proper signaling and plasticity.

### 4.2 Astrocytes

Astrocytes are crucial for maintaining water and neurotransmitter homeostasis, oxidative stress defense, tissue repair, and for regulating blood supply in an activity-dependent manner (Bélanger et al., 2011; Bordone et al., 2019; Barros and Weber, 2018). Additionally, astrocytes play a key role in synapse formation, maintenance, and remodeling. Their unique architecture and anatomical location enable astrocytes to mediate neurovascular coupling and regulate blood flow according to local metabolic demand (MacVicar and Newman, 2015; Mulligan and MacVicar, 2004). They form extensive and bidirectional contacts with blood vessels, allowing them to take up glucose and other metabolites from the bloodstream. They also secrete vasoactive molecules - such as prostaglandins, nitric oxide, and arachidonic acid - that modulate blood vessel diameter to influence cerebral perfusion (Gordon et al., 2007; Iadecola and Nedergaard, 2007). Structurally, astrocytes have two distinct types of processes: vascular endfeet which enwrap brain capillaries and arteries, and perisynaptic processes which ensheath synapses (Ko et al., 2023).

Astrocytic metabolism is tightly regulated by neuronal activity, particularly through extracellular K^+^ and glutamate. During action potential firing, neurons release K^+^ into the extracellular space (Somjen, 1979), activating the Na^+^/K^+^-ATPase in astrocytes (Grisar et al., 1979; Hajek et al., 1996) (Fig. 3). This decreases astrocytic ATP levels and activates glycolysis, finally increasing lactate production and release (Sokoloff et al., 1996), which - according to the ANLS hypothesis - can be taken up by neurons to fuel OXPHOS. Neuronal activity also elevates intracellular Na^+^ in astrocytes, but through a different mechanism: when synaptically released glutamate is cleared from the extracellular space, astrocytes take it up via Na^+^-dependent glutamate co-transporters, simultaneously transporting Na^+^ into astrocytes. Similarly to K^+^, increased Na^+^ levels activate the Na^+^/K^+^-ATPase (Denton et al., 1988). This again triggers glycolysis, thereby coupling glutamate uptake directly to glucose metabolism (Pellerin and Magistretti, 1994). In addition, glutamate itself can serve as energetic substrate, as it is converted to α-ketoglutarate and subsequently oxidized through the TCA cycle (Fonnum, 1968). Beyond these immediate metabolic effects, astrocytic uptake of glutamate and GABA also initiates the glutamate/GABA–glutamine cycle, which is essential for sustaining neurotransmission and preventing excitotoxicity. After uptake, glutamate and GABA are converted into glutamine by the glutamine synthetase, an enzyme exclusively expressed in astroglia and not in neurons. The newly synthesized glutamine is then exported from astrocytes and taken up by neurons, where it is reconverted into glutamate, and potentially GABA, thereby replenishing presynaptic neurotransmitter pools. Importantly, glutamate, GABA, and glutamine are all interconnected and can feed into the TCA cycle, providing additional energetic flexibility for both neurons and astrocytes (Andersen and Schousboe, 2023; Schousboe et al., 2013). Disruption of this cycle impairs neurotransmitter homeostasis and contributes to neurodegenerative pathologies, including tau-related disorders (Sidoryk-Węgrzynowicz et al., 2024).

**Fig. 3:**
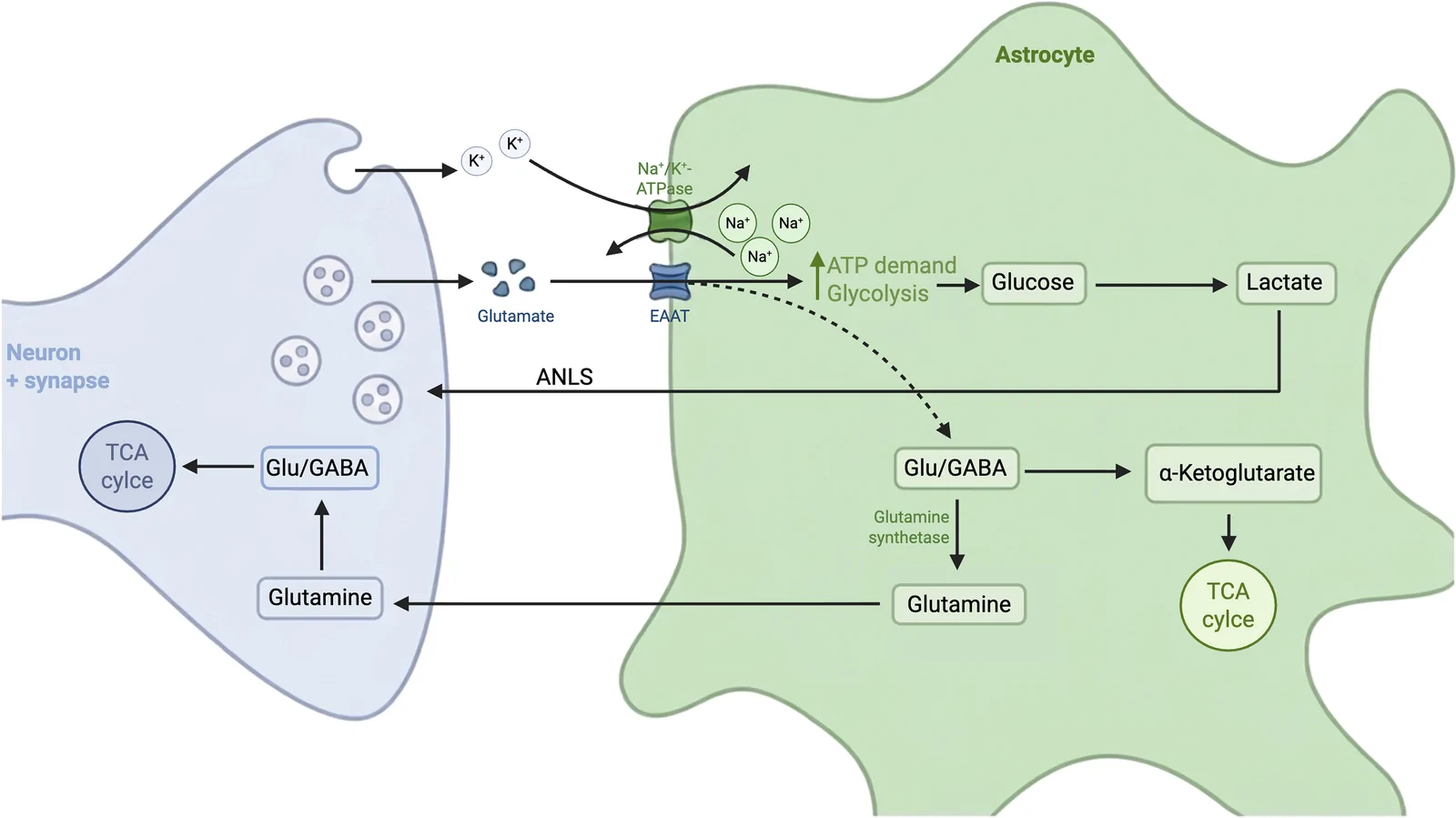
Activity-dependent astrocyte–neuron metabolic coupling. Neuronal action potential firing releases K^+^ and neurotransmitters into the extracellular space. Astrocytic uptake of K^+^ and Na^+^-dependent glutamate transport activates the Na^+^/K^+^-ATPase, increasing ATP demand and stimulating glycolysis. Astrocytes convert glucose to lactate, which can be shuttled to neurons, and metabolize glutamate via conversion to α-ketoglutarate. In parallel, glutamate and GABA are converted to glutamine by astrocyte-specific glutamine synthetase and recycled back to neurons to replenish neurotransmitter pools. Intermediates of the glutamate/GABA–glutamine cycle feed into the TCA cycle in both cell types, linking neurotransmission to energy metabolism. Created with BioRender.

Unlike neurons, astrocytes metabolize around 80% of glucose via glycolysis to produce pyruvate and lactate. This preference of glycolysis over oxidative metabolism is facilitated by a high astrocytic expression of glycolytic enzymes such as 6-phosphofructo-2-kinase/fructose-2,6-biphosphatase 3 and pyruvate dehydrogenase kinase 4 (PDK4), which are inhibited in neurons (Beard et al., 2022). Moreover, astrocytes express lactate dehydrogenase 5 which facilitates the conversion of pyruvate to lactate, whereas in neurons the expression of lactate dehydrogenase 1 favors the conversion of lactate to pyruvate, although net flux ultimately depends on substrate availability and cellular redox state (Magistretti and Allaman, 2018).

In the context of the ANLS, lipid droplets have emerged as an important component of neuron-glia metabolic coupling and neuronal protection against oxidative stress. Neuronal activity stimulates the transfer of peroxidized fatty acids from neurons to astrocytes, where they are stored in lipid droplets and subsequently metabolized, thereby protecting neurons from lipid-induced toxicity (Ioannou et al., 2019). These findings established lipid trafficking as an additional form of neuron-glia metabolic coupling that complements the exchange of energetic substrates such as lactate (Liu et al., 2017; Magistretti and Allaman, 2018). It is hypothesized that astrocytes metabolize lipid droplets during sleep, contributing to lipid clearance and mitochondrial function (Haynes et al., 2024). Sleep deprivation, in contrast, leads to lipid droplet accumulation in microglia which is associated with increased neuroinflammation (Marschallinger et al., 2020). These findings highlight the role of sleep in maintaining brain homeostasis and explain the link between sleep disorders and neurodegeneration. Lipid droplet transport relies on apolipoproteins such as ApoE and ApoD. Notably, the AD risk allele ApoE4 fails to efficiently accumulate and transport lipid droplets, thereby promoting neurodegeneration (Liu et al., 2017). In mice, the failure of astrocytes to efficiently degrade lipid droplets has been shown to cause AD-like symptoms. Specifically, impaired oxidative phosphorylation in astrocytes increases levels of fatty acids and acetyl-CoA, causing STAT3 acetylation, and activation of reactive astrocytes. These reactive astrocytes drive neurotoxicity through multiple mechanisms: (1) by increasing fatty acid oxidation and oxidative stress in neurons, (2) by activating microglia via IL-3 signaling, and (3) by inhibiting fatty acid synthesis needed for myelin production (Mi et al., 2023). Together, these findings underline the central role of astrocytes in energy metabolism, not only in supporting neuronal function, but also in protecting against inflammatory signaling and neurodegeneration.

### 4.3 Oligodendrocytes

Oligodendrocytes generate myelin sheaths that electrically insulate axons and thereby reduce the energetic cost of action potential propagation (Jastrowitz, 1870; Kuhn et al., 2019). The process of myelination is regulated by several factors, including Ca^2+^ signaling and neuronal activity (Kuhn et al., 2019). Beyond their insulating function, oligodendrocytes, similar to astrocytes, play a crucial role in axonal energy metabolism, as myelinated axons depend on local metabolic support to maintain long-range signal transmission. Oligodendrocytes supply neurons with energy substrates, most notably lactate, which is released via the monocarboxylate transporter MCT1 and taken up by axons to support ATP production. Disruption of MCT1-mediated substrate transfer leads to axonal degeneration, underscoring the essential role of oligodendrocyte-derived metabolic support for neuronal survival (Bercury and Macklin, 2015; Kuhn et al., 2019). In addition to lactate, oligodendrocytes can provide TCA-cycle intermediates to neurons, further contributing to the maintenance of neuronal energy homeostasis. Impairment of this metabolic coupling results in axonal dysfunction and promotes neurodegeneration (Fünfschilling et al., 2012).

Because myelination and neuronal support are highly energy-demanding processes, oligodendrocytes are particularly vulnerable to disturbances in energy metabolism. Accordingly, oligodendrocyte dysfunction is observed in several neurological disorders, including multiple sclerosis, schizophrenia, and AD (Kuhn et al., 2019). In multiple sclerosis, immune-mediated attacks on oligodendrocytes result in demyelination and secondary neuronal degeneration, making it the most prevalent autoimmune disease of the central nervous system (Binamé et al., 2021). In AD, oligodendrocyte degeneration is thought to arise as a secondary consequence of amyloid-β toxicity, further compromising axonal metabolic support and exacerbating neurodegeneration (Kuhn et al., 2019).

### 4.4 Microglia

Microglia, the macrophages of the central nervous system, are involved in blood vessel formation, neuronal maintenance, differentiation, and neuronal plasticity, but primarily regulate brain homeostasis by clearing the extracellular environment of microbes and dead or damaged cells (Kettenmann et al., 2011; Badanjak et al., 2021; Harry, 2013). Historically, microglia have been classified into the pro-inflammatory M1 and the anti-inflammatory M2 types. However, this binary classification is now considered an oversimplification, as microglial activation states exist along a dynamic spectrum that varies according to brain region, microenvironment, and disease context (Kwon and Koh, 2020). In neurodegeneration, distinct disease-associated microglial (DAM) states have been identified at sites of pathology, characterized by unique transcriptional signatures that do not conform to the classical M1/M2 framework (Keren-Shaul et al., 2017; Deczkowska et al., 2018). Increased microglial activation contributes to neuroinflammation, altered substrate utilization, and neuronal vulnerability in neurodegeneration (Badanjak et al., 2021).

From a metabolic perspective, resting microglia primarily rely on OXPHOS, while activated microglia primarily rely on aerobic glycolysis (Li et al., 2023). This metabolic switch is functionally relevant, as demonstrated in an LPS-induced PD model, where their inhibition by the glycolysis inhibitor 2-DG reduced neuroinflammation and the loss of dopaminergic neurons (Cheng et al., 2021). In AD, inhibition of glycolytic enzyme hexokinase-2 was found to increase microglial phagocytosis of amyloid-β (Leng et al., 2022), suggesting that metabolic modulation might enhance microglial function. In more advanced stages of AD, however, microglia often fail to clear the plaques (Rajendran and Paolicelli, 2018). This loss-of-function might also be associated with telomere shortening in the context of aging. Senescent cells release pro-inflammatory cytokines, finally causing accumulation of dysfunctional cells and molecules which microglia fail to efficiently clear (Rossiello et al., 2022). More specifically, microglia have been observed to release fragmented and dysfunctional mitochondria, triggering a pro-inflammatory response which activates A1 pro-inflammatory astrocytes, finally leading to neuronal death (Joshi et al., 2019).

## 5 Energy failure modes in neurodegeneration

Disruption of cellular energy metabolism is increasingly recognized across a broad spectrum of neurological disorders. While AD and PD serve as the principal disease models discussed in this review, similar metabolic disturbances have also been reported in Huntington's disease, amyotrophic lateral sclerosis, epilepsy, and inherited mitochondrial disorders such as Leigh syndrome (Table 1). These conditions differ substantially in their genetic and pathological origins but share common features including mitochondrial dysfunction, impaired substrate utilization, oxidative stress, and reduced metabolic flexibility. Together, they illustrate how disturbances in brain energy metabolism can contribute to neuronal vulnerability across diverse disease contexts.

**Table 1:** Metabolic dysfunction across neurological and neurodegenerative disorders.

Disease	Major metabolic alterations	Key cells/circuits	Evidence/models	Potential therapeutic strategies	References
Parkinson's disease (PD)	Complex I dysfunction, impaired mitophagy, altered mitochondrial dynamics	Dopaminergic neurons, astrocytes, microglia	Human genetics, imaging, postmortem tissue, animal and iPSC models	Mitophagy modulation, mitochondrial dynamics, metabolic modulation	(Walter et al., 2019; Murali Mahadevan et al., 2021; Hsieh et al., 2016; Boecker, 2023; Singh et al., 2021; Cai et al., 2019)

Alzheimer's disease (AD)	Cerebral glucose hypometabolism, brain insulin resistance, mitochondrial dysfunction	Neurons, astrocytes, microglia	Human FDG-PET, post-mortem tissue, animal models and iPSC models	Insulin signaling modulation, GLP-1 agonists, mitochondrial support	(Hoyer et al., 1991; Abolhassani et al., 2017; de la Monte and Wands, 2008; de la Monte, 2014; Shoshan-Barmatz et al., 2018; Csajbók and Tamás, 2016; Ghasemi et al., 2013; Ferreira et al., 2018; Cummings et al., 2026; Corcoran et al., 2026; Johri, 2021)

Huntington's disease (HD)	Impaired glucose utilization, elevated lactate metabolism, ETC dysfunction	Striatal neurons, hypothalamic circuits, astrocytes	Transgenic animals, patient tissue, cellular models	Mitochondrial quality control, metabolic support	(Hult et al., 2011; Jenkins et al., 2005; Gu et al., 1996)

Amyotrophic lateral sclerosis (ALS)	Mitochondrial dysfunction, hypermetabolism, glial metabolic dysfunction	Motor neurons, astrocytes, microglia	Human metabolic studies, SOD1 models	Metabolic support, mitochondrial targeting	(Liu et al., 2004; Vijayvergiya et al., 2005; Coussee et al., 2011; Dupuis et al., 2011; Bouteloup et al., 2009; Clement et al., 2003; Di Giorgio et al., 2007)

Epilepsy	Increased metabolic demand, oxidative stress	Neurons, astrocytes	Animal models, mitochondrial epilepsies, human studies	Ketogenic metabolism, antioxidant approaches	(Liang et al., 2000; Ben-Ari et al., 1980)

Leigh syndrome	Defective oxidative phosphorylation, lactic acidosis	Neurons, glia	Human genetics, patient studies, mitochondrial disease models	Metabolic support, mitochondrial-targeted therapies	(Marin et al., 2013; Dahl, 1998)

Charcot-Marie-Tooth (CMT)	Impaired mitochondrial dynamics, oxidative stress	Peripheral neurons, Schwann cells	Human genetics, patient studies, cellular and animal models	Restoration of mitochondrial dynamics, oxidative stress modulation	(Züchner et al., 2004; Niemann et al., 2009)

Comparative summary of the major metabolic defects, affected cell types, evidence, and therapeutic implications discussed in the review.

### 5.1 Parkinson's disease

Parkinson's disease (PD) is the second most common age-related neurodegenerative disease, affecting about 2–3% of the population above the age of 65 (Poewe et al., 2017). Clinically PD is characterized by motor symptoms including, resting tremor, rigidity and bradykinesia (Charcot, 1872; Parkinson, 1817). In addition, many patients present with a spectrum of non-motor symptoms such as hyposmia, cognitive deficits, depression, and rapid eye movement (REM) sleep behavior disorders, which often precede motor symptoms by years or decades (Li et al., 2024).

Pathologically, PD is defined as the progressive loss of dopaminergic (DA) neurons in the *substantia nigra pars compacta* (SN) (Bernheimer et al., 1973; Braak et al., 2003; Tretiakoff, 1919) and by the presence of Lewy bodies (Lewy, 1912), cytoplasmic aggregates primarily composed of α-synuclein (Poewe et al., 2017). The severity and cell-type specificity of this degeneration have prompted the concept of selective vulnerability of DA neurons in PD. Studies suggest that DA neurons are particularly susceptible to degeneration due to their excessive energetic demands, driven by dopamine synthesis and the maintenance of long, highly branched axonal projections (Braak and Del Tredici, 2009; Pissadaki and Bolam, 2013). In addition, DA neurons exhibit slow autonomous pacemaking activity characterized by broad action potentials, alongside a constant influx of calcium. This calcium influx further increases metabolic demand by requiring sustained ATP-dependent ion pumping, rendering DA neurons especially vulnerable compared to many other neighboring neuronal populations (Surmeier et al., 2010). Building on these intrinsic vulnerabilities, studies have identified additional molecular mechanisms that may further contribute to the selective susceptibility of DA neurons. By single-cell transcriptional profiling, Kamath et al. could identify the gene *AGTR1* to be strongly upregulated in PD patients' DA neurons compared to controls. AGTR1 was found to act on *TP53* and *NR2F2* induced stress pathways (Kamath et al., 2022). Interestingly, DA neurons expressing the vesicular glutamate transporter (VGLUT) - allowing them to release both dopamine and glutamate – appear to be more resilient to neurodegeneration (Buck et al., 2022). VGLUT is expressed in most DA neurons during their development but repressed in mature DA neurons. It may also contribute to the lower prevalence of PD in females, who express higher levels of VGLUT (Buck et al., 2022). However, its expression must be tightly regulated, as overexpression paradoxically triggers DA neuron loss (Buck et al., 2022). Another factor contributing to the selective vulnerability of DA neurons is their unusually high exposure to oxidative stress. Dopamine synthesis and metabolism can generate ROS by its labile catechol group which can auto-oxidize to quinone (Hastings and Zigmond, 1994), and H_2_O_2_ by the monoamine oxidase (Maker et al., 1981). This is further supported by studies showing that dopamine is toxic to neuronal cultures that are not co-cultured with astrocytes (Mena et al., 1997), again demonstrating the astrocytes' clearing function. Together, these intrinsic properties place DA neurons under exceptional bioenergetic and oxidative stress, rendering them particular susceptible to mitochondrial dysfunction.

The importance of mitochondrial dysfunction in PD was first highlighted by the observation that exposure to the toxin 1-methyl-4-phenyl-1,2,3,4-tetrahydropyridine (MPTP) induces an acute and irreversible Parkinsonian syndrome (Davis et al., 1979; Langston et al., 1983). Subsequent work demonstrated that MPTP is converted by monoamine oxidase to MPP+ (Singer et al., 1986) and taken up through dopamine transporters leading to accumulation in DA neurons (Javitch et al., 1985) where it inhibits complex I of the mitochondrial ETC, leading to ATP depletion and neuronal death (Nicklas et al., 1985). Similar findings were later observed in PD patients, where complex I deficiencies have been reported (Walter et al., 2019; González-Rodríguez et al., 2021). Moreover, overexpression of the NAD-dependent metabolic enzyme in Drosophila – which is necessary for complex I activity – improves neurodegeneration in PD-associated PINK1 mutants (Murali Mahadevan et al., 2021; Celardo et al., 2017). The complex I inhibitor rotenone induced the death of DA neurons, and experiments identified microtubule depolymerization as a potential underlying mechanism (Choi et al., 2011). Systemic deficiencies of complex I were observed in the striatum (Mizuno et al., 1989), platelet mitochondria (Parker et al., 1989), the SN (Schapira et al., 1989) and in skeletal muscle (Bindoff et al., 1989; Mizuno et al., 1989; Cardellach et al., 1993; Blin et al., 1994) of PD patients, although other studies reported normal complex I activity in the skeletal muscle (Anderson et al., 1993; Reichmann et al., 1994). The inhibited ETC also increases ROS production, causing dopamine auto-oxidation, inflammasome activation, and damage to the mitochondrial DNA (mtDNA) (Borsche et al., 2021). In PD, mtDNA is known to have reduced copy numbers and to contain deletions (Borsche et al., 2021). When in the cytosol, mtDNA is also considered a danger-associated molecular pattern (DAMP), which drives inflammation by activating the cGAS/STING pathway (West et al., 2015; Borsche et al., 2021). Defects in mitochondrial clearance which are observed in PD, especially in the PINK1 mutation, finally increase the number of dysfunctional mitochondria (Murali Mahadevan et al., 2021).

Although approximately 85–90% of PD cases are sporadic, with no known cause, around 10–15% are attributed to pathogenic genetic variants (Lesage and Brice, 2012; Coppedè, 2012). Five genes—*SNCA, LRRK2, PARK2 (parkin), PINK1,* and *PARK7 (DJ-1)*—account for the majority of monogenic PD cases (Nuytemans et al., 2010). Despite their diverse primary functions, these genes converge mechanistically on mitochondrial integrity, energy production, and quality control (Grünewald et al., 2019). Mutations in *SNCA* promote α-synuclein aggregation, which disrupts mitochondrial membranes, respiratory chain activity, and proteostasis (Polymeropoulos et al., 1997; Krüger et al., 1998). *LRRK2* mutations impair mitochondrial dynamics, trafficking, and microtubule-based transport, thereby compromising the spatial distribution of energy supply within neurons (Funayama et al., 2002; Zimprich et al., 2004).

The E3 ubiquitin ligase Parkin (Imai et al., 2000; Shimura et al., 2000; Zhang et al., 2000) interacts with Pink1 in mitochondrial turnover (Exner et al., 2007; Deng et al., 2008; Poole et al., 2008; Park et al., 2009) and quality control. Upon depolarization of the mitochondrial membrane, the cytoplasmic Parkin binds to the mitochondrial mitofusin (Mfn)2. Pink1 phosphorylates Mfn2 and thus promotes Parkin-mediated ubiquitination (Chen and Dorn, 2013). This selective recruitment of Parkin leads to a targeted elimination of mitochondria, or autophagy (Narendra et al., 2008, Narendra et al., 2009). Disruption of Parkin is connected to increased ROS, contributing to the formation of the NLRP3 inflammasome, which is involved in the loss of DA neurons (Panicker et al., 2022). ROS also induces dopamine auto-oxidation and damages the mitochondrial genome (Borsche et al., 2021), which in turn causes dysregulation and increased ROS production. Pink1 is also implicated in mitochondrial trafficking along microtubules in axons by forming a complex with the atypical GTPase Miro and the adaptor protein Milton, both of which are localized in the outer mitochondrial membrane and responsible for microtubule binding (Weihofen et al., 2009). Mitochondrial motility is an important aspect of mitochondrial turnover dynamics, especially in highly polarized cells such as neurons. DJ-1 has RNA-binding activity, specifically to some RNAs involved in glutathione metabolism, and dissociates from RNA after oxidative stress (van der Brug et al., 2008). As damaged mitochondria with an impaired ETC generate ROS, DJ-1 acts in a parallel, antioxidant pathway to the Pink1/Parkin pathway to control autophagy (Thomas et al., 2011; de Castro et al., 2010; Giordano et al., 2014). Despite their diverse molecular functions, PD-associated genes converge on a limited set of pathways regulating mitochondrial respiration, mitophagy, lysosomal-autophagic function, ROS homeostasis, immune signaling, and intracellular energy distribution. This convergence further supports mitochondrial dysfunction as a central driver of selective DA vulnerability.

Mitochondrial dysfunction is not restricted to idiopathic PD but is also observed across genetically defined and atypical parkinsonian syndromes. Defects involving nuclear-encoded or mitochondrial respiratory-chain proteins can produce parkinsonism as part of broader neurological phenotypes, supporting mitochondrial impairment as a shared pathogenic mechanism across clinically heterogeneous parkinsonian disorders (Legati and Ghezzi, 2023).

In summary, these findings support the brain energy metabolism hypothesis of PD etiology, classifying the death of DA neurons in the SN as a malfunction of mitochondrial energy generation. Given the intra- and intercellular orchestration of brain energy metabolism described above, the future directions in PD research need to consider the heterogeneous brain architecture and develop methods to investigate cell-cell interactions in a systematic manner. Although there are a couple of PD animal models available (Antony et al., 2011), they do not yet account for the disease complexity and often ignore energetic aspects.

### 5.2 Alzheimer's disease

Alzheimer's disease (AD) is the most common cause of dementia and affects around 50 million people worldwide (Breijyeh and Karaman, 2020). It is characterized by progressive cognitive dysfunction with widespread degeneration and loss of cellular mass in cortical and subcortical areas (Wenk, 2006). This neurodegeneration is characterized pathologically by intracellular neurofibrillary tangles consisting of phosphorylated Tau and extracellular amyloid-β plaques (Breijyeh and Karaman, 2020). A small percentage of early-onset cases can be attributed to genetic mutations, e.g. the genes *APP*, *PSEN1*, and *PSEN2*, which alter amyloid processing and promote disease development. Large GWAS studies have identified further AD susceptibility loci (Medway and Morgan, 2014). Several of these genes are linked to cellular energy metabolism and mitochondrial function. Among them, *ApoE4* represents the strongest genetic risk factor and has been associated with altered brain glucose metabolism and mitochondrial dysfunction (Wu et al., 2018; Budny et al., 2025). Further susceptibility loci, including *ATP5H/KCTD2,* have also been related to mitochondrial energy production (Boada et al., 2014).

Accordingly, mitochondrial dysfunction is also a major hallmark of AD. It involves imbalanced Ca^2+^ concentrations, increased oxidative stress and dysregulated apoptosis (Shoshan-Barmatz et al., 2018). Mitochondrial oxidative stress causes hyperphosphorylation of tau (Melov et al., 2007), thereby contributing to the formation of the characteristic neurofibrils. Additionally, overexpression of the mitochondrial voltage-dependent anion channel 1 (VDAC1) is associated with neuronal loss in AD patients (Shoshan-Barmatz et al., 2018). Impaired mitochondrial fission and fusion, as well as mutations in the mitochondrial genome were also found in AD patients (Wang et al., 2009; Santoro et al., 2010). Furthermore, amyloid-β interferes with mitochondrial function (Casley et al., 2002; Manczak et al., 2006), impacts glucose metabolism, spatially correlates with the Warburg effect (Xie et al., 2013; Vlassenko et al., 2010), and directly localizes to mitochondria (Shoshan-Barmatz et al., 2018). On the level of astrocytes, oxidative stress reduces neuronal viability in AD (Allaman et al., 2010) and induces a reduction of neurovascular coupling by retraction of astrocytic endfeet from capillaries (Merlini et al., 2011). As mentioned above, impaired OXPHOS in astrocytic mitochondria can cause fatty acid accumulation, activating inflammatory pathways which increase oxidative stress in neurons, as shown in an AD mouse model (Mi et al., 2023).

Epidemiological studies found that type 2 diabetes mellitus represents a significant risk factor for AD (Bosco et al., 2011), also demonstrated by an impaired insulin-regulated glucose homeostasis within the brain of AD patients (Haley et al., 2006; Duarte et al., 2012). Brain glucose metabolism is decreased in early stages of the disease (Hoyer et al., 1991; Abolhassani et al., 2017) and impairments in cerebral glucose utilization and energy metabolism precede initial stages of cognitive impairment (Hoyer et al., 1991; Iwangoff et al., 1980).

In line with this, AD is also referred to as “type 3 diabetes” due to an insulin insensitivity (resistance) and deficiency in the brain (de la Monte and Wands, 2008; de la Monte, 2014). The exact mechanism of brain insulin resistance in AD remains elusive (Biessels and Despa, 2018). However, some molecular mechanisms have been identified: In addition to synthesis in the pancreas, insulin can be synthesized at specific brain regions (Devaskar et al., 1993; Devaskar et al., 1994). Here, it modulates hippocampal neurons (Van Der Heide et al., 2005) and plays a role in regulating synaptic activity (Ghasemi et al., 2013). Insulin is also involved in the modulation of neurotransmitter channel activity, brain cholesterol synthesis and mitochondrial function (Abolhassani et al., 2017). Insulin and insulin-like-growth-factor signaling impairments generate deficits in energy metabolism and increase oxidative stress, mitochondrial dysfunction, activation of proinflammatory cytokines and increased APP expression (de la Monte and Wands, 2008). Insulin resistance in the brain impairs both amyloid-β processing and clearance, as well as tau protein expression and phosphorylation (Nguyen et al., 2020). This perspective of AD being caused by an underlying insulin resistance and insensitivity has been increasingly supported by various findings in the last years. For example, rats showed AD-like pathology when exposed to streptozotocin, a potent generator of ROS that selectively targets insulin-producing cells (de la Monte and Wands, 2008). In contrast, elevated brain insulin levels and nasal insulin administration can improve memory and cognitive function (Csajbók and Tamás, 2016; Ghasemi et al., 2013) by preventing synapse loss and preserving surface insulin receptor levels (Ferreira et al., 2018). Similarly, the insulin sensitizer metformin is currently being evaluated in a phase III clinical trial expected to be completed in 2026 (Cummings et al., 2026). Growing evidence therefore supports insulin-related pathways as therapeutic targets in AD. In particular, glucagon-like peptide-1 (GLP-1) receptor agonists, which are widely used to treat type 2 diabetes by enhancing and prolonging insulin signaling, have attracted considerable attention because GLP-1 receptors are also expressed in the hippocampus. Preclinical and early clinical studies have reported beneficial effects of liraglutide, dulaglutide, and semaglutide on cognitive function and disease-related pathology, although clinical evidence remains limited and several trials are still ongoing (Corcoran et al., 2026; Cummings et al., 2026).

## 6 Broader spectrum of metabolic dysfunction in neurodegeneration and neurological disorders

Metabolic dysfunctions similar to those described in PD and AD are also observed in other neurodegenerative and neurological diseases, further highlighting the central role of impaired brain energy metabolism in neurodegeneration, as illustrated below for selected pathologies.

### 6.1 Huntington's disease

Huntington's disease (HD) is a progressive neurodegenerative disorder characterized by motor disturbance, cognitive decline, and psychiatric manifestations (Martin and Gusella, 1986) and exhibits a strong association with disrupted energy metabolism. For example, transgenic mouse models overexpressing mutant HTT exhibit impaired glucose metabolism and elevated lactate levels in the hypothalamus, indicating defective pyruvate utilization and mitochondrial dysfunction (Jenkins et al., 2005). This dysregulation is accompanied by a pronounced insulin and leptin resistance, suggesting systemic dysregulation of energy homeostasis (Hult et al., 2011). Consistent with these findings, post-mortem analyses of HD patient tissue have revealed marked deficiencies in ETC complexes II, III, and IV within mitochondria isolated from the caudate nucleus (Gu et al., 1996). Abnormal mitochondrial pathology, including enlarged mitochondria has also been reported in lymphoblasts from HD patients, with homozygous individuals displaying a more severe phenotype. Together with evidence of impaired oxidative function in skeletal muscle from both asymptomatic mutation carriers and symptomatic patients, these findings support a causal contribution of mitochondrial dysfunction in HD pathogenesis (Saft et al., 2005). In addition to impaired bioenergetics, mitochondria in HD display impaired calcium handling, altered intracellular trafficking, and disrupted network dynamics (Panov et al., 2002). Mutant HTT protein promotes excessive mitochondrial fission through increased Drp1 activity, resulting in mitochondrial fragmentation and reduced energetic efficiency, which may contribute to early weight loss in HD patients (Djoussé et al., 2002). Furthermore, HTT localizes glycolytic enzymes to vesicles during fast axonal transport to locally supply ATP to molecular motors. Thus, disruption of this process by mutant HTT is likely to impair axonal transport and mitochondrial renewal, reinforcing the link between mitochondrial bioenergetics and HD pathology (Zala et al., 2013).

### 6.2 Amyotrophic lateral sclerosis

Amyotrophic lateral sclerosis (ALS) comprises a group of fatal neurodegenerative disorders characterized by the progressive loss of upper and lower motor neurons, resulting in muscle weakness and paralysis. Approximately 5–10% of ALS cases are familial, with mutations in the superoxide dismutase 1 (SOD1) gene accounting for ∼20% of familial ALS and ∼ 1–2% of sporadic cases (Wijesekera and Nigel Leigh, 2009). Although SOD1 is a key antioxidant enzyme involved in ROS detoxification, increasing evidence supports a toxic gain-of-function mechanism for mutant SOD1, rather than simple loss of enzymatic activity (Shaw, 2005). Mitochondrial dysfunction and impaired energy metabolism are central features of ALS pathology. Like DA neurons, motor neurons have exceptionally high energetic demands due to their large size, long axons, and sustained firing activity, rendering them particularly vulnerable to bioenergetic stress. Mutant SOD1 has been shown to associate with mitochondria, where it disrupts ETC activity, increases ROS production, and impairs ATP generation, ultimately leading to energetic failure and neurodegeneration (Liu et al., 2004; Vijayvergiya et al., 2005; Coussee et al., 2011). ALS progression is further shaped by non–cell-autonomous mechanisms. Astrocytes and microglia expressing mutant SOD1 exhibit impaired metabolic and antioxidant support, altered glutamate handling, and increased inflammatory signaling, which exacerbate neuronal energy failure (Clement et al., 2003; Di Giorgio et al., 2007). At the systemic level, ALS patients frequently display hypermetabolism and weight loss, both of which correlate with faster disease progression and reduced survival, highlighting the relevance of whole-body energy imbalance to disease outcome (Dupuis et al., 2011; Bouteloup et al., 2009). Beyond SOD1, additional ALS-associated genes such as TDP-43, FUS, and C9orf72 converge on pathways regulating mitochondrial function, RNA metabolism, and cellular energy homeostasis (Wang et al., 2016; Mehta et al., 2021). Together, these findings reinforce impaired brain energy metabolism as a unifying contributor to motor neuron vulnerability in ALS.

### 6.3 Primary mitochondrial encephalopathies

Although epilepsy is conventionally not classified as a primary neurodegenerative disorder, recurrent seizures impose exceptionally high metabolic demands and can cause mitochondrial oxidative stress and neuronal injury. After seizure induction with the excitotoxin kainite in rats, the seizures could be associated with mitochondrial superoxide production (Liang et al., 2000; Patel et al., 2001). This indicates the large activity dependent energy demand. Recently, it was shown that the scaffolding protein RGS14 can reduce this superoxide production (Harbin et al., 2023). Some rare forms of epilepsy, such as mitochondrial encephalomyopathy, lactic acidosis and stroke-like episodes (MELAS), as well as myoclonic epilepsy with ragged red fibers (MERRF), are directly associated with mitochondrial DNA mutations (Rahman, 2012). Mitochondrial encephalopathy leads to progressive myoclonic epilepsy, cognitive impairment, and dementia. Biochemically, mitochondrial myopathies can be divided into defects of substrate utilization, oxidation-phosphorylation coupling, and the ETC (DiMauro et al., 1985). MERRF symptoms include epileptic seizures, myoclonus, spasticity, ataxia, dementia and loss of sensation. In most cases, the mitochondrial tRNA gene and thus all mitochondrial gene transcripts are affected (Blakely et al., 2013; Monden et al., 2013). Epilepsy's relation to energy metabolism is further illustrated by some treatment options which include a ketonic diet which provides ketones and fatty acids as alternative metabolites to the glycolytic substrates, improving the mitochondrial redox status (Jarrett et al., 2008), and melatonin injection which reduces seizure-induced cell death in mice by its radical scavenging properties (Tan et al., 1998).

Leigh syndrome is a rare disease characterized by demyelination, vascular proliferation and gliosis, caused by deficiencies in mitochondrial ATP production. The disorder is genetically heterogeneous and can be caused by mutations in both nuclear and mitochondrial genes. Among the most common genetic causes are mutations in the complex I subunit *NDUFS8*, and the mitochondrially encoded subunit of the ATP synthase, *MT-ATP6*, both of which disrupt OXPHOS and cellular energy homeostasis (Dahl, 1998; Brunetti et al., 2021; Lorenz et al., 2017). In some cases, the patients show lactic acidosis due to the accumulation of pyruvate, which is unable to run through the TCA cycle (Marin et al., 2013). The excessive pyruvate is subsequently either converted into alanine or lactic acid. The excess of both is accumulating in the body and disturbs metabolic homeostasis. Additionally, mitochondria of a patient with the Leigh syndrome have been found to have abnormal cristae structures (Siegmund et al., 2018), showing that they are metabolically dysregulated.

Charcot-Marie-Tooth (CMT) disease is one of the most common inherited peripheral neuropathies. Some of the CMT-associated genes encode mitochondrial proteins, such as mitofusin 2 (Mfn2) (Züchner et al., 2004) and ganglioside-induced differentiation associated protein 1 (GDAP1) (Niemann et al., 2005). Mfn2 is a key regulator of mitochondrial fusion and mutations lead to dominant inherited CMT (Züchner et al., 2004). For GDAP1, 53 different pathogenic mutations have been characterized (Cassereau et al., 2011). Recessively inherited disease mutants show reduced mitochondrial fragmentation, whereas dominantly inherited disease mutants interfere with mitochondrial fusion, resulting in a disturbed mitochondrial membrane potential and increased ROS levels (Niemann et al., 2009).

Beyond mechanistic studies, increasing evidence from patient-derived samples and clinical imaging supports a link between bioenergetic dysfunction and disease progression (Grünewald et al., 2026; Borsche et al., 2021; Salmon et al., 2024). Alterations in mitochondrial respiration, ATP production, mitophagy, and oxidative metabolism have been reported in peripheral blood cells, cerebrospinal fluid, fibroblasts, and other patient-derived tissues from individuals with AD, PD, HD, and ALS (Pickett et al., 2018; Mortiboys et al., 2010; Hsieh et al., 2016; Saft et al., 2005). In several cases, metabolic abnormalities have been associated with clinical features or disease progression, including cognitive decline, motor impairment, and hypermetabolism (Dupuis et al., 2011; Bouteloup et al., 2009; Borsche et al., 2021), suggesting that metabolic phenotypes may provide clinically relevant information on disease vulnerability and progression. Importantly, metabolic abnormalities can be detected before substantial neuronal loss becomes apparent. In AD, for example, FDG-PET hypometabolism is detectable during prodromal and even preclinical stages and can be associated with subsequent cognitive decline or conversion to dementia (Salmon et al., 2024; Chételat et al., 2020). Similarly, mitochondrial and bioenergetic abnormalities have been reported in genetically at-risk individuals and patient-derived cellular models of neurodegenerative disease (Saft et al., 2005; Borsche et al., 2021). These observations raise the possibility that bioenergetic profiling could serve not only as a mechanistic tool but also as a biomarker of disease progression and treatment response.

The growing interest in metabolic biomarkers has been accompanied by substantial advances in the experimental approaches available to study brain energy metabolism. Neuroimaging and patient-derived disease models provide complementary levels of information, ranging from regional metabolic activity in vivo to cell type-specific bioenergetic dysfunction, while biochemical, metabolic, and multi-omics approaches enable increasingly detailed characterization of substrate utilization and mitochondrial function. Together, these approaches provide a framework for investigating how metabolic alterations emerge, evolve, and contribute to disease progression (Chételat et al., 2020).

## 7 Methods for studying brain metabolism

One of the most widely used approaches is [18F]fluoro-deoxy-glucose positron emission tomography (FDG-PET), which measures regional cerebral glucose utilization in vivo. Following injection, the glucose analogue 18F-FDG is transported through glucose transporters and phosphorylated, allowing the assessment of tissue glucose uptake and glycolytic activity, although its signal should not be equated directly with complete glycolytic flux or neuronal glucose metabolism because it integrates contributions from multiple cell types. FDG-PET is particularly well established for detecting characteristic cerebral hypometabolic patterns in AD and may also reveal disease-associated network-level metabolic patterns in PD and atypical parkinsonian disorders (Peralta et al., 2019; Meles et al., 2017; Salmon et al., 2024). Related PET approaches, including amyloid-PET and tau-PET, provide complementary information on disease pathology and can be integrated with metabolic imaging to improve diagnostic accuracy (Chételat et al., 2020). In PD, metabolic imaging is often combined with imaging of DA dysfunction using 18F-DOPA PET or dopamine transporter single-photon emission computed tomography (DAT-SPECT) (Garnett et al., 1983; Stoessl et al., 2014; Poewe et al., 2017).

Magnetic resonance spectroscopy (MRS) complements PET by providing non-invasive measurements of brain metabolites such as lactate, glutamate, glutamine, *N*-acetylaspartate, and other compounds involved in neuronal and glial metabolism. Because MRS can be performed in conjunction with conventional MRI, it enables simultaneous assessment of structural and metabolic alterations and has become an important tool for studying neurodegenerative disorders and mitochondrial diseases (Öz et al., 2014; Salmon et al., 2024).

Finally, patient-derived induced pluripotent stem cell (iPSC) models have emerged as a powerful platform for investigating cell type-specific metabolic dysfunction. Fibroblasts obtained from patients can be reprogrammed into iPSCs using Yamanaka factors (Takahashi and Yamanaka, 2006; Takahashi et al., 2007) and subsequently differentiated into disease-relevant cell types such as DA neurons, astrocytes, oligodendrocytes, or microglia. These models enable detailed bioenergetic profiling, mitochondrial analyses, and multi-omic investigations while retaining the patient's genetic background, thereby bridging mechanistic studies and human disease (Mlody et al., 2016; Weykopf et al., 2019; Babu and Snyder, 2023).

Collectively, molecular imaging, metabolic spectroscopy, and patient-derived cellular models provide complementary biomarkers of brain energy metabolism that can be used for diagnosis, patient stratification, monitoring disease progression, and assessing therapeutic responses.

## 8 Therapeutic restoration of metabolic resilience

As described above, many neurodegenerative disorders are characterized by early and progressive failure of brain energy metabolism, reflected by reduced glucose utilization, altered mitochondrial dynamics, and disrupted metabolic coupling between neurons and glia cells. However, most currently approved therapies primarily target neurotransmission or protein aggregation and only indirectly affect cellular bioenergetics. In PD, dopamine replacement strategies such as L-DOPA, dopamine agonists, and MAO-B inhibitors improve motor symptoms but do not address the underlying decline in mitochondrial ATP production (Poewe et al., 2017). Notably, MAO-B inhibitors reduce the formation of toxic dopamine metabolites, thereby lowering mitochondrial oxidative stress and partially preserving energy metabolism (Tan et al., 2022). In AD, cholinesterase inhibitors and NMDA receptor antagonists modulate synaptic signaling but remain ineffective once neuronal energy demand can no longer be met due to mitochondrial dysfunction and neuronal loss (Morris et al., 2021). Symptomatic benefits do not necessarily correct the underlying bioenergetic abnormalities that may contribute to disease progression. Beyond neurotransmitter-based therapies, recent disease-modifying strategies have focused on reducing pathological protein aggregation. In AD, monoclonal antibodies targeting amyloid-β, including lecanemab and aducanumab, have demonstrated the ability to reduce plaque formation, while therapeutic efforts in PD increasingly aim to limit α-synuclein accumulation (Cummings et al., 2023). However, many of these interventions remain in clinical development, and their long-term efficacy and impact on disease progression remain uncertain. Taken together, the partial success of therapies targeting neurotransmission or protein aggregation reflects the multifactorial nature of neurodegeneration, in which metabolic failure, mitochondrial dysfunction, and impaired intercellular support precede overt neuronal loss. Therapeutic strategies aimed at restoring energy homeostasis may therefore complement disease-specific approaches by acting at a more integrative level of cellular function.

Given that mitochondria are the primary source of neuronal ATP, therapeutic strategies increasingly aim to restore mitochondrial function and turnover. Drugs targeting the mitochondrial transport protein Miro1 enhance mitophagy and promote the replacement of dysfunctional mitochondria, thereby improving oxidative phosphorylation capacity (Capriglia et al., 2023). Similarly, inhibition of excessive mitochondrial fission through Drp1 inhibitors counteracts mitochondrial fragmentation, preserves respiratory efficiency, and reduces neurodegeneration in experimental models (Johri, 2021; Reddy et al., 2011; Mangrulkar et al., 2023). However, these compounds remain at an early stage of development and have not yet advanced to clinical evaluation. Moreover, as discussed above, mitochondrial fission can play both protective and pathological roles depending on cellular context, and therapeutic modulation of fission pathways requires careful contextualization, as broad inhibition may impair mitochondrial quality control or prove deleterious in certain cellular or disease settings.

Targeting mitochondrial substrate flux represents another approach to rebalance energy metabolism. Mitochondria-targeted antioxidants aim to preserve mitochondrial bioenergetic capacity by limiting oxidative damage via ROS generation. MitoQ, a ubiquinone derivative that accumulates in the mitochondrial matrix, scavenges ROS and thereby supports ETC function. In preclinical models, MitoQ attenuates oxidative stress and minimizes neurodegenerative pathology (Johri, 2021). Importantly, MitoQ readily crosses both the BBB and the mitochondrial membrane. Recent evidence further suggests that MitoQ may improve mitochondrial dynamics by suppressing Drp1-mediated fission and promoting OPA1-mediated fusion (Wu et al., 2025). Similarly, the coenzyme Q10 analogue idebenone reduces oxidative stress, promotes mitochondrial quality control, and improves motor and cognitive performance in experimental models (Yan et al., 2022; Zhang et al., 2025b). Despite encouraging preclinical findings, convincing evidence for consistent clinical benefit remains limited, and clinical trials have yielded mixed results (Zhang et al., 2025b). In the case of MitoQ, no significant symptomatic improvement has yet been demonstrated in neurodegenerative disease (Shinn and Lagalwar, 2021). Similarly, a recent clinical study failed to detect significant benefits in healthy older adults, despite promising effects previously observed in mouse models (Murray et al., 2026). While MitoQ has shown more encouraging results in certain cardiometabolic conditions (Pang et al., 2025), these findings highlight the challenges of translating metabolic interventions from experimental systems to human neurological disease.

Several repurposed drugs have also been shown to increase ATP availability. Terazosin, originally used to treat hypertension and prostate hyperplasia, activates phosphoglycerate kinase 1 (PGK1), enhancing glycolytic flux and elevating ATP levels in both neuronal and patient-derived PD models (Cai et al., 2019). Moreover, in LRRK2 PD models, terazosin has been shown to reduce α-synuclein accumulation and neuronal death, supporting its potential to minimize PD-associated pathologies (Capriglia et al., 2023). Clinical findings remain mixed, with epidemiological studies finding no clear reduction in PD risk among terazosin-treated individuals compared with controls (Sasane et al., 2021), whereas other studies reported beneficial associations—including epidemiological evidence for reduced PD risk (Simmering et al., 2022), increased blood ATP levels in patients with PD (Schultz et al., 2022), and enhanced parameters of energy metabolism in healthy older individuals (Schultz et al., 2025). Adding to the mixed clinical findings, terazosin exerts pleiotropic and systemic effects, reflecting a mechanism of action that is not restricted to neuronal energy metabolism, which may complicate its translational application and necessitates careful evaluation of dosing and target engagement (McHale-Owen et al., 2025).

Another example of metabolic intervention through drug repurposing is ursodeoxycholic acid (UDCA), originally approved for the treatment of primary biliary cholangitis. In patient-derived fibroblasts carrying the PD-associated *LRRK2* G2019S mutation, UDCA improves ATP levels and mitochondrial performance, linking bile acid signaling to neuronal energy homeostasis (Mortiboys et al., 2015). These findings are supported by beneficial outcomes in animal models (Abdelkader et al., 2016), suggesting that modulation of mitochondrial metabolism may translate across experimental systems. Early clinical studies have demonstrated acceptable safety and reported potential improvements in PD-associated outcomes, although these findings should be interpreted cautiously given the small patient cohorts and limited number of studies available (Sathe et al., 2020; Payne et al., 2023; Capriglia et al., 2023).

More recently, the anti-tumor drug lapatinib ditosylate has been identified as a modulator of mitochondrial metabolism through a distinct upstream mechanism. It alters estrogen-related receptor α (ERRα) signaling and downstream MPC-1 expression, thereby reshaping mitochondrial substrate utilization and energy metabolism. In experimental models, this metabolic reprogramming is associated with improved mitochondrial function, reduced neuroinflammation, and neuroprotective effects, highlighting transcriptional control of mitochondrial metabolism as an additional therapeutic entry point in neurodegenerative disease (Mangrulkar et al., 2023). Consistent with this concept, lapatinib increased dopaminergic neuron survival in rotenone-induced PD models (Mansour et al., 2023) and improved AD-like pathology in rat models (Mansour et al., 2021). However, evidence for lapatinib in neurodegeneration remains limited to preclinical studies, and clinical trials in patients with neurodegenerative diseases have not yet been conducted.

Regular physical activity exerts anti-inflammatory effects, reduces cardiovascular risk, and improves cognitive performance, in part by enhancing mitochondrial function and metabolic resilience in the brain (Johri, 2021). Additionally, accumulating evidence indicates that dietary interventions substantially influence the progression of neurodegeneration, with the ketogenic diet representing a primary example of metabolic modulation. Under prolonged fasting or ketogenic conditions, ketone bodies partially replace glucose as the primary cerebral energy substrate (López-Ojeda and Hurley, 2023). This metabolic state can be induced through a high-fat, low-carbohydrate diet or by supplementation with medium-chain fatty acids or ketone salts (Johri, 2021). Moreover, when insulin levels are low, ketone bodies can partially replace glucose as an energy source for neurons (Jensen et al., 2020). In experimental models, ketogenic diets improve mitochondrial function through the upregulation of key regulators of mitochondrial biogenesis and efficiency, including PGC-1α, SIRT3, and UCP2 (Hasan-Olive et al., 2019). Despite encouraging preclinical findings, the clinical evidence supporting ketogenic interventions in neurodegenerative disease remains limited. Existing studies are often constrained by small sample sizes, short follow-up periods, and a lack of data on long-term safety and efficacy. Furthermore, adherence to ketogenic diets can be challenging in everyday clinical practice, and inter-individual variability in treatment response remains poorly understood (Rubio et al., 2025). Additionally, dietary supplements like vitamin D, melatonin, and nicotinamide riboside are the subject of numerous studies and positively affect mitochondria. Resveratrol and melatonin, e.g., activate the peroxisome proliferator-activated receptor-γ-coactivator (PGC)-1α and decrease neurodegeneration. Interestingly, PGC-1α is also a target of the diabetes drug pioglitazone which improves cognitive function in dementia (Heneka et al., 2015; Mangrulkar et al., 2023). Taken together, physical activity, ketogenic strategies, and metabolic supplementation converge on mitochondrial biogenesis, efficiency, and redox balance, reinforcing brain energy metabolism as a central and modifiable determinant of neuronal resilience.

Nevertheless, most evidence remains preclinical and the translation of metabolic interventions into effective clinical treatments has proven challenging. Translation is complicated by considerable inter-patient heterogeneity, differences in disease stage, limited BBB penetration, and the complexity of neurodegenerative disease mechanisms. Consequently, future therapeutic strategies will likely require both improved patient stratification and more targeted delivery approaches, such as nanoparticle-based drug delivery systems, to maximize treatment efficacy (Capriglia et al., 2023; Zhang et al., 2025a; Zhang et al., 2025b; Shinn and Lagalwar, 2021).

## 9 Discussion

Brain energy metabolism is increasingly recognized not as a collection of independent metabolic pathways, but as a dynamic, multicellular network in which neuronal and glial processes couple electrical activity to substrate availability, mitochondrial function, and inflammatory signaling. Disruption at any level of this network can propagate metabolic stress across cell types and contribute to neurodegeneration.

A recurring theme emerging from recent work is the activity-dependent role of astrocytes as metabolic integrators. Astrocytic responses to extracellular K^+^ and glutamate link synaptic activity directly to glycolysis, lactate production, and neurotransmitter recycling (Magistretti and Allaman, 2015; Pellerin and Magistretti, 1994; Mächler et al., 2016). While the ANLS provides an influential framework for understanding this coupling, accumulating evidence indicates that lactate utilization by neurons is highly context-dependent (Dienel, 2017, Dienel, 2019; Díaz-García et al., 2017; Díaz-García and Yellen, 2018). Taken together, biosensor-based studies emphasizing neuronal glycolysis coexist with in vivo evidence consistent with lactate transfer and neuronal lactate utilization, suggesting that substrate preference is shaped by factors such as activity pattern, developmental stage, metabolic state, and disease context. Rather than a universal rule, astrocyte–neuron metabolic coupling appears to operate as a flexible, state-dependent system.

Mitochondria emerge as a critical hub where multiple metabolic and pathological signals converge. Neurons with high energetic demands, such as *substantia nigra* dopaminergic neurons, exhibit several intrinsic features that increase their metabolic vulnerability, including extensive axonal arborization, autonomous Ca^2+^ pacemaking, and dopamine-associated oxidative stress (Surmeier et al., 2010). In this setting, mitochondrial dynamics and quality control become decisive. Balanced fusion, fission, transport, and mitophagy are essential to sustain ATP production and limit ROS accumulation. Importantly, recent work has revealed that mitochondrial fission is not a monolithic process (Wang et al., 2023; Boecker, 2023): peripheral and midzone fission events serve distinct functions, and broad inhibition of fission can be deleterious. The controversy surrounding LRRK2-dependent regulation of fission exemplifies how pathogenic stressors can produce divergent mitochondrial phenotypes depending on cellular context, disease stage, and metabolic state.

Beyond neurons, glial contributions to energy homeostasis are increasingly recognized as disease-modifying. Astrocytes and oligodendrocytes contribute to neuronal and axonal metabolic support through substrate provision and regulation of lipid metabolism, while astrocytic lipid droplet formation and fatty-acid metabolism can influence cellular responses to metabolic and oxidative stress. Failure of these support mechanisms, whether through impaired lipid handling, disrupted myelin-associated metabolic coupling, or reactive glial states, can amplify neuronal energy failure. Microglia further shape this landscape through immunometabolic reprogramming, linking inflammation to altered substrate utilization and mitochondrial stress.

Across neurodegenerative diseases, these mechanisms converge on a common outcome: progressive loss of metabolic flexibility. In AD, early cerebral glucose hypometabolism and brain insulin resistance compromise neuronal energy supply well before overt neurodegeneration. In PD, genetic and environmental insults converge on mitochondrial bioenergetics and quality control. In Huntington's disease and ALS, impaired glucose utilization, mitochondrial dysfunction, and glial metabolic failure contribute to selective neuronal vulnerability. Together, these observations support the view that neurodegeneration arises not from a single metabolic defect, but from the failure of adaptive metabolic coordination across cell types (Table 1).

Despite substantial progress, several major questions remain unresolved. First, the extent to which neurons rely on glucose, lactate, ketone bodies, or lipid-derived substrates under different physiological and pathological conditions remains debated. In particular, the context-dependency of the ANLS highlights the need to reconcile findings obtained from biosensor-based imaging, isotope tracing, and in vivo metabolic measurements. Second, the temporal relationship between metabolic dysfunction and neurodegeneration remains unclear. While impaired glucose utilization, mitochondrial dysfunction, and insulin resistance are frequently observed before neuronal loss, it remains uncertain which metabolic alterations represent primary disease drivers and which arise secondarily during disease progression. Importantly, metabolic changes that are often interpreted as pathological, including increased glycolysis, lactate production, lipid droplet formation, and mitochondrial biogenesis, may in some contexts represent adaptive responses to energetic stress rather than primary drivers of neurodegeneration. More broadly, it remains unclear which substrates are preferentially utilized under different physiological and pathological conditions, how substrate selection changes during disease progression, and at what stage metabolic dysfunction emerges relative to neuronal degeneration. Longitudinal studies combining metabolic imaging approaches such as FDG-PET and MR spectroscopy with molecular and clinical readouts will be important for establishing the temporal relationship between metabolic alterations and neurodegeneration. Additional challenges arise from the increasing recognition of cell-type-specific metabolism. Astrocytic lipid droplet formation, oligodendrocyte-mediated metabolic support, and microglial immunometabolic reprogramming have emerged as important regulators of neuronal resilience, yet their precise contributions to disease progression remain incompletely understood.

Overcoming these limitations will require experimental systems that more faithfully recapitulate human disease biology. A major limitation of the current evidence base is that mechanistic insights into brain energy metabolism are derived predominantly from cellular and animal models, whereas human studies generally provide lower-resolution measurements of metabolic alterations. Although patient-derived cells and clinical imaging increasingly bridge this gap, it remains challenging to establish whether metabolic phenotypes observed in experimental models accurately reflect metabolic processes occurring in the human brain in vivo. This limitation is particularly relevant in neurodegenerative disease, where acute toxin-based models, monocultures, young animals, and models carrying familial disease mutations often fail to reproduce the slow progression, cellular diversity, and metabolic heterogeneity observed in sporadic disease. Furthermore, important modifiers such as aging, sex, vascular comorbidities, and systemic metabolic dysfunction are frequently underrepresented. These limitations are particularly relevant for metabolic studies, because substrate availability, mitochondrial function, and glial metabolic states are strongly influenced by aging, systemic metabolism, vascular function, and cellular interactions. As a result, findings obtained in experimental systems do not always translate into clinically meaningful therapeutic benefits, highlighting the need for models that more accurately reflect the metabolic landscape of human disease.

Addressing these challenges will require experimental systems that better recapitulate the cellular and metabolic complexity of human neurodegenerative disease. Patient-derived multicellular iPSC models, organoids, and co-culture systems provide opportunities to investigate neuron–glia metabolic interactions in a human genetic background, while stable-isotope tracing and genetically encoded metabolic sensors can resolve substrate utilization and metabolic responses at cellular resolution. Emerging approaches including spatial transcriptomics, spatial metabolomics, single-cell multi-omics, and multimodal imaging may further reveal how metabolic states are organized across cell types and change throughout disease progression. Integrating these approaches with longitudinal clinical cohorts and metabolic imaging could help distinguish early adaptive responses from progressive metabolic failure, identify disease-stage-specific vulnerabilities, and establish which metabolic alterations are causally linked to neuronal degeneration. Importantly, patient-derived models also provide an opportunity to investigate metabolic heterogeneity between individuals and disease subtypes, providing a basis for identifying patient-specific metabolic vulnerabilities and developing more targeted therapeutic strategies.

Ultimately, the emerging picture is not one of a single defective metabolic pathway, but of a progressive loss of metabolic flexibility and intercellular coordination. Understanding when and where this flexibility breaks down, and which metabolic responses are compensatory versus pathogenic, may be critical for identifying metabolic vulnerabilities that can be therapeutically targeted before irreversible neuronal loss occurs.

## 10 Methods

This article represents a narrative review of the literature for which literature searches were conducted up to spring 2026. Studies were selected based on their relevance to cell-type-specific brain metabolism, mechanistic insight, evidence from human disease, or historical importance. Studies addressing systemic metabolism without a clear connection to brain function or neurodegeneration were generally excluded. Relevant publications were identified primarily through searches of the PubMed database conducted throughout the preparation of the manuscript using combinations of terms including *brain energy metabolism*, *neuroenergetics*, *astrocyte*, *neuron*, *microglia*, *oligodendrocyte*, *mitochondria*, *mitophagy*, *glucose metabolism*, *lactate*, *ketone bodies*, *Alzheimer's disease*, *Parkinson's disease*, *Huntington's disease*, and *amyotrophic lateral sclerosis*. Additional studies were identified through backward and forward citation tracking of relevant review articles and primary research studies. Primary literature was prioritized for specific mechanistic and experimental claims, while review articles were used to provide broader conceptual context and synthesize established areas of the field. Influential historical studies and recent advances were incorporated to provide both historical context and an up-to-date perspective. As a narrative review, the literature search was not conducted according to a predefined systematic-review protocol; therefore, no formal study selection or screening criteria, risk-of-bias assessment, or meta-analysis was performed.

## Declaration of generative AI in the manuscript preparation process

ChatGPT was used to support the generation of Fig. 1. The final figure was reviewed, redone or edited by the authors.

## CRediT authorship contribution statement

**Carmen Maria Lahr:** Writing – review & editing, Writing – original draft, Visualization, Conceptualization. **Elle Wilson:** Writing – review & editing, Writing – original draft, Visualization, Conceptualization. **Melanie Sengupta:** Writing – original draft. **Michela Bernini:** Writing – original draft, Conceptualization. **Alexander Skupin:** Writing – review & editing, Writing – original draft, Supervision, Project administration, Funding acquisition, Conceptualization.

## Declaration of competing interest

None.

## Data Availability

No data was used for the research described in the article.

## References

[bb0005] Abdelkader Noha F., Safar Marwa M., Salem Hesham A. (2016). Ursodeoxycholic acid ameliorates apoptotic cascade in the rotenone model of Parkinson’s disease: modulation of mitochondrial perturbations. Mol. Neurobiol..

[bb0010] Abolhassani Nona, Leon Julio, Sheng Zijing (2017). Molecular pathophysiology of impaired glucose metabolism, mitochondrial dysfunction, and oxidative DNA damage in Alzheimer’s disease brain. Mech. Ageing Dev..

[bb0015] Allaman Igor, Gavillet Mathilde, Bélanger Mireille (2010). Amyloid-beta aggregates cause alterations of astrocytic metabolic phenotype: impact on neuronal viability. J. Neurosci..

[bb0020] Allen Nicola J., Barres Ben A. (2009). Neuroscience: glia - more than just brain glue. Nature.

[bb0025] Almeida A., Moncada S., Bolaños J.P. (2004). Nitric oxide switches on glycolysis through the AMP protein kinase and 6-phosphofructo-2-kinase pathway. Nat. Cell Biol..

[bb0030] Ames A. (2000). CNS energy metabolism as related to function. Brain Res. Brain Res. Rev..

[bb0035] Andersen Jens V., Schousboe Arne (2023). Milestone review: metabolic dynamics of glutamate and GABA mediated neurotransmission — the essential roles of astrocytes. J. Neurochem..

[bb0040] Anderson J.J., Bravi D., Ferrari R. (1993). No evidence for altered muscle mitochondrial function in Parkinson’s disease. J. Neurol. Neurosurg. Psychiatry.

[bb0045] Antony Paul M.A., Diederich Nico J., Balling Rudi (2011). Parkinson’s disease mouse models in translational research. Mamm. Genome.

[bb0050] Arena Giuseppe, Sharma K., Agyeah G., Krüger R., Grünewald A., Fitzgerald J.C. (2022). Neurodegeneration and neuroinflammation in Parkinson’s disease: a self-sustained loop. Curr. Neurol. Neurosci. Rep..

[bb0055] Ashrafi Ghazaleh, Wu Zhuhao, Farrell Ryan J., Ryan Timothy A. (2017). GLUT4 mobilization supports energetic demands of active synapses. Neuron.

[bb0060] Attwell D., Laughlin S.B. (2001). An energy budget for signaling in the grey matter of the brain. J. Cereb. Blood Flow Metab..

[bb0065] Babu Mohan, Snyder Michael (2023). Multi-omics profiling for health. Mol. Cell. Proteomics.

[bb0070] Badanjak Katja, Fixemer Sonja, Smajić Semra, Skupin Alexander, Grünewald Anne (2021). The contribution of microglia to neuroinflammation in Parkinson’s disease. Int. J. Mol. Sci..

[bb0075] Bak Lasse K., Walls Anne B., Schousboe Arne, Waagepetersen Helle S. (2018). Astrocytic glycogen metabolism in the healthy and diseased brain. J. Biol. Chem..

[bb0080] Barros L.F., Weber B. (2018). CrossTalk proposal: an important astrocyte-to-neuron lactate shuttle couples neuronal activity to glucose utilisation in the brain. J. Physiol..

[bb0085] Beard Elidie, Lengacher Sylvain, Dias Sara, Magistretti Pierre J., Finsterwald Charles (2022). Astrocytes as key regulators of brain energy metabolism: new therapeutic perspectives. Front. Physiol..

[bb0090] Bélanger Mireille, Allaman Igor, Magistretti Pierre J. (2011). Brain energy metabolism: focus on astrocyte-neuron metabolic cooperation. Cell Metab..

[bb0095] Ben-Ari Y., Tremblay E., Ottersen O.P., Meldrum B.S. (1980). The role of epileptic activity in hippocampal and ‘remote’ cerebral lesions induced by kainic acid. Brain Res..

[bb0100] Bercury Kathryn K., Macklin Wendy B. (2015). Dynamics and mechanisms of CNS myelination. Dev. Cell.

[bb0105] Bernheimer H., Birkmayer W., Hornykiewicz O., Jellinger K., Seitelberger F. (1973). Brain dopamine and the syndromes of Parkinson and Huntington. Clinical, morphological and neurochemical correlations. J. Neurol. Sci..

[bb0110] Bertholet A.M., Delerue T., Millet A.M. (2016). Mitochondrial fusion/fission dynamics in neurodegeneration and neuronal plasticity. Neurobiol. Dis..

[bb0115] Biessels Geert Jan, Despa Florin (2018). Cognitive decline and dementia in diabetes mellitus: mechanisms and clinical implications. Nat. Rev. Endocrinol..

[bb0120] Binamé Fabien, Pham-Van Lucas D., Bagnard Dominique (2021). Manipulating oligodendrocyte intrinsic regeneration mechanism to promote remyelination. Cell. Mol. Life Sci..

[bb0125] Bindoff L.A., Birch-Machin M., Cartlidge N.E., Parker W.D., Turnbull D.M. (1989). Mitochondrial function in Parkinson’s disease. Lancet (London, England).

[bb0130] Blakely Emma L., Yarham John W., Alston Charlotte L. (2013). Pathogenic mitochondrial tRNA point mutations: nine novel mutations affirm their importance as a cause of mitochondrial disease. Hum. Mutat..

[bb0135] Blin O., Desnuelle C., Rascol O. (1994). Mitochondrial respiratory failure in skeletal muscle from patients with Parkinson’s disease and multiple system atrophy. J. Neurol. Sci..

[bb0140] Boada M., Antúnez C., Ramírez-Lorca R. (2014). ATP5H/KCTD2 locus is associated with Alzheimer’s disease risk. Mol. Psychiatry.

[bb0145] Boecker C. Alexander (2023). The role of LRRK2 in intracellular organelle dynamics. J. Mol. Biol..

[bb0150] Bonvento Gilles, Bolaños Juan P. (2021). Astrocyte-neuron metabolic cooperation shapes brain activity. Cell Metab..

[bb0155] Bordone Melina Paula, Salman Mootaz M., Titus Haley E. (2019). The energetic brain – a review from students to students. J. Neurochem..

[bb0160] Borsche Max, Pereira Sandro L., Klein Christine, Grünewald Anne (2021). Mitochondria and Parkinson’s disease: clinical, molecular, and translational aspects. J. Parkinsons Dis..

[bb0165] Bosco Domenico, Fava Antonietta, Plastino Massimiliano, Montalcini Tiziana, Pujia Arturo (2011). Possible implications of insulin resistance and glucose metabolism in Alzheimer’s disease pathogenesis. J. Cell. Mol. Med..

[bb0170] Boumezbeur Fawzi, Petersen Kitt, Cline Gary (2010). The contribution of blood lactate to brain energy metabolism in humans measured by dynamic C-13 nuclear magnetic resonance spectroscopy. J. Neurosci..

[bb0175] Bouteloup C., Desport J.C., Clavelou P. (2009). Hypermetabolism in ALS patients: an early and persistent phenomenon. J. Neurol..

[bb0180] Bouzier-Sore Anne-Karine, Voisin Pierre, Bouchaud Véronique, Bezancon Eric, Franconi Jean-Michel, Pellerin Luc (2006). Competition between glucose and lactate as oxidative energy substrates in both neurons and astrocytes: a comparative NMR study. Eur. J. Neurosci..

[bb0185] Braak Heiko, Del Tredici Kelly (2009). Neuroanatomy and pathology of sporadic Parkinson’s disease. Adv. Anat. Embryol. Cell Biol..

[bb0190] Braak Heiko, Del Tredici Kelly, Rüb Udo, de Vos Rob A.I., Jansen Steur Ernst N.H., Braak Eva (2003). Staging of brain pathology related to sporadic Parkinson’s disease. Neurobiol. Aging.

[bb0195] Breijyeh Zeinab, Karaman Rafik (2020). Comprehensive review on Alzheimer’s disease: causes and treatment. Molecules (Basel, Switzerland).

[bb0200] Brown Angus M., Ransom Bruce R. (2007). Astrocyte glycogen and brain energy metabolism. Glia.

[bb0205] Brunetti Dario, Dykstra Werner, Le Stephanie, Zink Annika, Prigione Alessandro (2021). Mitochondria in neurogenesis: implications for mitochondrial diseases. Stem Cells (Dayton, Ohio).

[bb0210] Buck Silas A., Quincy Erickson-Oberg M., Bhatte Sai H. (2022). Roles of VGLUT2 and dopamine/glutamate co-transmission in selective vulnerability to dopamine neurodegeneration. ACS Chem. Neurosci..

[bb0215] Budny Vanessa, Ruminot Iván, Wybitul Maha, Treyer Valerie, Barros L. Felipe, Tackenberg Christian (2025). Fueling the brain - the role of apolipoprotein E in brain energy metabolism and its implications for Alzheimer’s disease. Transl. Psychiatry.

[bb0220] Cai Rong, Zhang Yu, Simmering Jacob E. (2019). Enhancing glycolysis attenuates Parkinson’s disease progression in models and clinical databases. J. Clin. Invest..

[bb0225] Calì Corrado, Tauffenberger Arnaud, Magistretti Pierre (2019). The strategic location of glycogen and lactate: from body energy reserve to brain plasticity. Front. Cell. Neurosci..

[bb0230] Camandola Simonetta, Mattson Mark P. (2017). Brain metabolism in health, aging, and neurodegeneration. EMBO J..

[bb0235] Capriglia Francesco, Burgess Toby, Bandmann Oliver, Mortiboys Heather (2023). Clinical trial highlights: modulators of mitochondrial function. J. Parkinsons Dis..

[bb0240] Cardellach F., Martí M.J., Fernández-Solá J. (1993). Mitochondrial respiratory chain activity in skeletal muscle from patients with Parkinson’s disease. Neurology.

[bb0245] Casley C.S., Canevari L., Land J.M., Clark J.B., Sharpe M.A. (2002). Beta-amyloid inhibits integrated mitochondrial respiration and key enzyme activities. J. Neurochem..

[bb0250] Cassereau Julien, Chevrollier Arnaud, Bonneau Dominique (2011). A locus-specific database for mutations in GDAP1 allows analysis of genotype-phenotype correlations in Charcot-Marie-Tooth diseases type 4A and 2K. Orphanet J. Rare Dis..

[bb0255] Cavanagh J.B. (1999). Corpora-amylacea and the family of polyglucosan diseases. Brain Res. Brain Res. Rev..

[bb0260] Celardo Ivana, Lehmann Susann, Costa Ana C., Loh Samantha H.Y., Miguel Martins L. (2017). dATF4 regulation of mitochondrial folate-mediated one-carbon metabolism is neuroprotective. Cell Death Differ..

[bb0265] Charcot, Jean-Martin (1825–-1893) Auteur du texte (1872). https://www.buecher.de/shop/buecher/leons-sur-les-maladies-du-systme-nerveux-faites--la-salptrires-1872-1873/charcot-jean-martin/products_products/detail/prod_id/59553448/.

[bb0270] Chen Yun, Dorn Gerald W. (2013). PINK1-phosphorylated mitofusin 2 is a Parkin receptor for culling damaged mitochondria. Science (New York, N.Y.).

[bb0275] Cheng Junjie, Zhang Rong, Xu Zhirou (2021). Early glycolytic reprogramming controls microglial inflammatory activation. J. Neuroinflammation.

[bb0280] Chételat Gaël, Arbizu Javier, Barthel Henryk (2020). Amyloid-PET and 18F-FDG-PET in the diagnostic investigation of Alzheimer’s disease and other dementias. Lancet Neurol..

[bb0285] Choi Won-Seok, Palmiter Richard D., Xia Zhengui (2011). Loss of mitochondrial complex I activity potentiates dopamine neuron death induced by microtubule dysfunction in a Parkinson’s disease model. J. Cell Biol..

[bb0290] Clement A.M., Nguyen M.D., Roberts E.A. (2003). Wild-type nonneuronal cells extend survival of SOD1 mutant motor neurons in ALS mice. Science (New York, N.Y.).

[bb0295] Cogliati Sara, Frezza Christian, Soriano Maria Eugenia (2013). Mitochondrial cristae shape determines respiratory chain supercomplexes assembly and respiratory efficiency. Cell.

[bb0300] Coppedè Fabio (2012). Genetics and epigenetics of Parkinson’s disease. ScientificWorldJournal.

[bb0305] Corcoran Eve, Kettlety Michael, Mogul Urwa, Azah Jennifer Ndiforngwah, Cork Simon C. (2026). The effects of GLP-1 receptor agonists on Alzheimer’s pathophysiology: a systematic review. Mol. Cell. Neurosci..

[bb0310] Cotter David G., André d’Avignon D., Wentz Anna E., Weber Mary L., Crawford Peter A. (2011). Obligate role for ketone body oxidation in neonatal metabolic homeostasis. J. Biol. Chem..

[bb0315] Coussee Evelyne, De Smet Patrick, Bogaert Elke (2011). G37R SOD1 mutant alters mitochondrial complex I activity, Ca(2+) uptake and ATP production. Cell Calcium.

[bb0320] Csajbók Éva A., Tamás Gábor (2016). Cerebral cortex: a target and source of insulin?. Diabetologia.

[bb0325] Cummings Jeffrey L., Leisgang Osse Amanda M., Kinney Jefferson W. (2023). Alzheimer’s disease: novel targets and investigational drugs for disease modification. Drugs.

[bb0330] Cummings Jeffrey L., Zhou Yadi, Yang Yuxin (2026). Alzheimer’s disease drug development pipeline: 2026. Alzheimers Dement. (New York, N. Y.).

[bb0335] Cunnane Stephen C. (2018). Ketones, omega-3 fatty acids and the Yin-Yang balance in the brain: insights from infant development and Alzheimer’s disease, and implications for human brain evolution. OCL.

[bb0340] Cunnane Stephen C., Courchesne-Loyer Alexandre, St-Pierre Valérie (2016). Can ketones compensate for deteriorating brain glucose uptake during aging? Implications for the risk and treatment of Alzheimer’s disease. Ann. N. Y. Acad. Sci..

[bb0345] Cunnane Stephen C., Trushina Eugenia, Morland Cecilie (2020). Brain energy rescue: an emerging therapeutic concept for neurodegenerative disorders of ageing. Nat. Rev. Drug Discov..

[bb0350] Dahl H.H. (1998). Getting to the nucleus of mitochondrial disorders: identification of respiratory chain-enzyme genes causing Leigh syndrome. Am. J. Hum. Genet..

[bb0355] Davis G.C., Williams A.C., Markey S.P. (1979). Chronic parkinsonism secondary to intravenous injection of meperidine analogues. Psychiatry Res..

[bb0360] Dawson Ted M., Dawson Valina L. (2003). Molecular pathways of neurodegeneration in Parkinson’s disease. Science (New York, N.Y.).

[bb0365] de Castro Inês Pimenta, Martins L. Miguel, Tufi Roberta (2010). Mitochondrial quality control and neurological disease: an emerging connection. Expert Rev. Mol. Med..

[bb0370] de la Monte Suzanne M. (2014). Type 3 diabetes is sporadic Alzheimer’s disease: mini-review. Eur. Neuropsychopharmacol..

[bb0375] de la Monte Suzanne M., Wands Jack R. (2008). Alzheimer’s disease is type 3 diabetes–evidence reviewed. J. Diabetes Sci. Technol. (Online).

[bb0380] Deczkowska Aleksandra, Keren-Shaul Hadas, Weiner Assaf, Colonna Marco, Schwartz Michal, Amit Ido (2018). Disease-associated microglia: a universal immune sensor of neurodegeneration. Cell.

[bb0385] Deng Hansong, Dodson Mark W., Huang Haixia, Guo Ming (2008). The Parkinson’s disease genes Pink1 and Parkin promote mitochondrial fission and/or inhibit fusion in drosophila. Proc. Natl. Acad. Sci. USA.

[bb0390] Denton R.M., Rutter G.A., Midgley P.J., McCormack J.G. (1988). Effects of Ca2+ on the activities of the calcium-sensitive dehydrogenases within the mitochondria of mammalian tissues. J. Cardiovasc. Pharmacol..

[bb0395] Devaskar Sherin U., Farouk Sadiq H., Holtzclaw Lynne, George Mark (1993). The developmental pattern of rabbit brain insulin and insulin-like growth factor receptor expression. Brain Res..

[bb0400] Devaskar S.U., Giddings S.J., Rajakumar P.A., Carnaghi L.R., Menon R.K., Zahm D.S. (1994). Insulin gene expression and insulin synthesis in mammalian neuronal cells. J. Biol. Chem..

[bb0405] Di Giorgio Francesco Paolo, Carrasco Monica A., Siao Michelle C., Maniatis Tom, Eggan Kevin (2007). Non-cell autonomous effect of glia on motor neurons in an embryonic stem cell-based ALS model. Nat. Neurosci..

[bb0410] Díaz-García Carlos Manlio, Yellen Gary (2018). Neurons rely on glucose rather than astrocytic lactate during stimulation. J. Neurosci. Res..

[bb0415] Díaz-García Carlos Manlio, Mongeon Rebecca, Lahmann Carolina, Koveal Dorothy, Zucker Hannah, Yellen Gary (2017). Neuronal stimulation triggers neuronal glycolysis and not lactate uptake. Cell Metab..

[bb0420] Dienel Gerald A. (2017). Lack of appropriate stoichiometry: strong evidence against an energetically important astrocyte–neuron lactate shuttle in brain. J. Neurosci. Res..

[bb0425] Dienel Gerald A. (2019). Brain glucose metabolism: integration of energetics with function. Physiol. Rev..

[bb0430] DiMauro S., Bonilla E., Zeviani M., Nakagawa M., DeVivo D.C. (1985). Mitochondrial myopathies. Ann. Neurol..

[bb0435] Djoussé L., Knowlton B., Cupples L.A., Marder K., Shoulson I., Myers R.H. (2002). Weight loss in early stage of Huntington’s disease. Neurology.

[bb0440] Dorn Gerald W. (2023). Reversing dysdynamism to interrupt mitochondrial degeneration in amyotrophic lateral sclerosis. Cells.

[bb0445] Duarte Ana I., Moreira Paula I., Oliveira Catarina R. (2012). Insulin in central nervous system: more than just a peripheral hormone. J. Aging Res..

[bb0450] Dupuis Luc, Pradat Pierre-François, Ludolph Albert C., Loeffler Jean-Philippe (2011). Energy metabolism in amyotrophic lateral sclerosis. Lancet Neurol..

[bb0455] Eberhardt Emily L., Ludlam Anthony V., Tan Zhenyu, Cianfrocco Michael A. (2020). Miro: a molecular switch at the center of mitochondrial regulation. Protein Sci..

[bb0460] Exner Nicole, Treske Bettina, Paquet Dominik (2007). Loss-of-function of human PINK1 results in mitochondrial pathology and can be rescued by Parkin. J. Neurosci..

[bb0465] Faria-Pereira Andreia, Morais Vanessa A. (2022). Synapses: the brain’s energy-demanding sites. Int. J. Mol. Sci..

[bb0470] Ferreira Eloise, Bignoux Monique J., Otgaar Tyrone C. (2018). LRP/LR specific antibody IgG1-iS18 impedes neurodegeneration in Alzheimer’s disease mice. Oncotarget.

[bb0475] Fonnum F. (1968). The distribution of glutamate decarboxylase and aspartate transaminase in subcellular fractions of rat and guinea-pig brain. Biochem. J..

[bb0480] Funayama Manabu, Hasegawa Kazuko, Kowa Hisayuki, Saito Masaaki, Tsuji Shoji, Obata Fumiya (2002). A new locus for Parkinson’s disease (PARK8) maps to chromosome 12p11.2-Q13.1. Ann. Neurol..

[bb0485] Fünfschilling Ursula, Supplie Lotti M., Mahad Don (2012). Glycolytic oligodendrocytes maintain myelin and long-term axonal integrity. Nature.

[bb0490] Garcia Guadalupe C., Bartol Thomas M., Phan Sébastien (2019). Mitochondrial morphology provides a mechanism for energy buffering at synapses. Sci. Rep..

[bb0495] Garnett E.S., Firnau G., Nahmias C. (1983). Dopamine visualized in the basal ganglia of living man. Nature.

[bb0500] Ghasemi Rasoul, Haeri Ali, Dargahi Leila, Mohamed Zahurin, Ahmadiani Abolhassan (2013). Insulin in the brain: sources, localization and functions. Mol. Neurobiol..

[bb0505] Giordano Samantha, Darley-Usmar Victor, Zhang Jianhua (2014). Autophagy as an essential cellular antioxidant pathway in neurodegenerative disease. Redox Biol..

[bb0510] González-Rodríguez Patricia, Zampese Enrico, Stout Kristen A. (2021). Disruption of mitochondrial complex I induces progressive parkinsonism. Nature.

[bb0515] Gordon Grant R.J., Mulligan Sean J., MacVicar Brian A. (2007). Astrocyte control of the cerebrovasculature. Glia.

[bb0520] Grisar T., Frere J.M., Franck G. (1979). Effect of K+ ions on kinetic properties of the (Na+, K+)-ATPase (EC 3.6.1.3) of bulk isolated glial cells, perikarya and synaptosomes from rabbit brain cortex. Brain Res..

[bb0525] Grünewald Anne, Kumar Kishore R., Sue Carolyn M. (2019). New insights into the complex role of mitochondria in Parkinson’s disease. Prog. Neurobiol..

[bb0530] Grünewald Anne, Knab Felix, Zimmermann Milan (2026). Blood mtDNA markers of mitochondrial subtype and early-onset Parkinson’s disease biology. Brain J. Neurol..

[bb0535] Gu M., Gash M.T., Mann V.M., Javoy-Agid F., Cooper J.M., Schapira A.H. (1996). Mitochondrial defect in Huntington’s disease caudate nucleus. Ann. Neurol..

[bb0540] Hajek I., Subbarao K.V., Hertz L. (1996). Acute and chronic effects of potassium and noradrenaline on Na+, K+-ATPase activity in cultured mouse neurons and astrocytes. Neurochem. Int..

[bb0545] Haley Andreana P., Knight-Scott Jack, Simnad Virginia I., Manning Carol A. (2006). Increased glucose concentration in the hippocampus in early Alzheimer’s disease following oral glucose ingestion. Magn. Reson. Imaging.

[bb0550] Hall C.N., Klein-Flugge M.C., Howarth C., Attwell D. (2012). Oxidative phosphorylation, not glycolysis, powers presynaptic and postsynaptic mechanisms underlying brain information processing. J. Neurosci..

[bb0555] Haque Md Ezazul, Akther Mahbuba, Jakaria Md, Kim In-Su, Azam Shofiul, Choi Dong-Kug (2020). Targeting the microglial NLRP3 inflammasome and its role in Parkinson’s disease. Mov. Disord..

[bb0560] Harbin N.H., Lustberg D.J., Hurst C. (2023). RGS14 limits seizure-induced mitochondrial oxidative stress and pathology in hippocampus. Neurobiol. Dis..

[bb0565] Harry G. Jean (2013). Microglia during development and aging. Pharmacol. Ther..

[bb0570] Hasan-Olive Md Mahdi, Lauritzen Knut H., Ali Mohammad, Rasmussen Lene Juel, Storm-Mathisen Jon, Bergersen Linda H. (2019). A ketogenic diet improves mitochondrial biogenesis and bioenergetics via the PGC1α-SIRT3-UCP2 axis. Neurochem. Res..

[bb0575] Hastings T.G., Zigmond M.J. (1994). Identification of catechol-protein conjugates in neostriatal slices incubated with [3H]dopamine: impact of ascorbic acid and glutathione. J. Neurochem..

[bb0580] Haynes Paula R., Pyfrom Elana S., Li Yongjun (2024). A neuron–glia lipid metabolic cycle couples daily sleep to mitochondrial homeostasis. Nat. Neurosci..

[bb0585] Heneka Michael T., Fink Anne, Doblhammer Gabriele (2015). Effect of pioglitazone medication on the incidence of dementia. Ann. Neurol..

[bb0590] Herrero-Mendez Angel, Almeida Angeles, Fernández Emilio, Maestre Carolina, Moncada Salvador, Bolaños Juan P. (2009). The bioenergetic and antioxidant status of neurons is controlled by continuous degradation of a key glycolytic enzyme by APC/C–Cdh1. Nat. Cell Biol..

[bb0595] Holman Geoffrey D. (2020). Structure, function and regulation of mammalian glucose transporters of the SLC2 family. Pflugers Arch. - Eur. J. Physiol..

[bb0600] Hoyer S., Nitsch R., Oesterreich K. (1991). Predominant abnormality in cerebral glucose utilization in late-onset dementia of the Alzheimer type: a cross-sectional comparison against advanced late-onset and incipient early-onset cases. J. Neural Transm. Park. Dis. Dement. Sect..

[bb0605] Hsieh Chung-Han, Shaltouki Atossa, Gonzalez Ashley E. (2016). Functional impairment in miro degradation and mitophagy is a shared feature in familial and sporadic Parkinson’s disease. Cell Stem Cell.

[bb0610] Hult Sofia, Soylu Rana, Björklund Tomas (2011). Mutant Huntingtin causes metabolic imbalance by disruption of hypothalamic neurocircuits. Cell Metab..

[bb0615] Hyder Fahmeed, Rothman Douglas L., Bennett Maxwell R. (2013). Cortical energy demands of signaling and nonsignaling components in brain are conserved across mammalian species and activity levels. Proc. Natl. Acad. Sci. USA.

[bb0620] Iadecola Costantino, Nedergaard Maiken (2007). Glial regulation of the cerebral microvasculature. Nat. Neurosci..

[bb0625] Imai Y., Soda M., Takahashi R. (2000). Parkin suppresses unfolded protein stress-induced cell death through its E3 ubiquitin-protein ligase activity. J. Biol. Chem..

[bb0630] Ioannou Maria S., Jackson Jesse, Sheu Shu-Hsien (2019). Neuron-astrocyte metabolic coupling protects against activity-induced fatty acid toxicity. Cell.

[bb0635] Iwangoff P., Armbruster R., Enz A., Meier-Ruge W. (1980). Glycolytic enzymes from human autoptic brain cortex: normal aged and demented cases. Mech. Ageing Dev..

[bb0640] Iwata Ryohei, Casimir Pierre, Erkol Emir (2023). Mitochondria metabolism sets the species-specific tempo of neuronal development. Science.

[bb0645] Jarrett Stuart G., Milder Julie B., Liang Li-Ping, Patel Manisha (2008). The ketogenic diet increases mitochondrial glutathione levels. J. Neurochem..

[bb0650] Jastrowitz M. (1870). Studien über die Encephalitis und Myelitis des ersten Kindesalters. Arch. Psychiatr. Nervenkr..

[bb0655] Javitch J.A., D’Amato R.J., Strittmatter S.M., Snyder S.H. (1985). Parkinsonism-inducing neurotoxin, N-methyl-4-phenyl-1,2,3,6 -tetrahydropyridine: uptake of the metabolite N-methyl-4-phenylpyridine by dopamine neurons explains selective toxicity. Proc. Natl. Acad. Sci. USA.

[bb0660] Jenkins Bruce G., Andreassen Ole A., Dedeoglu Alpaslan (2005). Effects of CAG repeat length, HTT protein length and protein context on cerebral metabolism measured using magnetic resonance spectroscopy in transgenic mouse models of Huntington’s disease. J. Neurochem..

[bb0665] Jensen Nicole Jacqueline, Wodschow Helena Zander, Nilsson Malin, Rungby Jørgen (2020). Effects of ketone bodies on brain metabolism and function in neurodegenerative diseases. Int. J. Mol. Sci..

[bb0670] Johri Ashu (2021). Disentangling mitochondria in Alzheimer’s disease. Int. J. Mol. Sci..

[bb0675] Joshi Amit U., Minhas Paras S., Liddelow Shane A. (2019). Fragmented mitochondria released from microglia trigger A1 astrocytic response and propagate inflammatory neurodegeneration. Nat. Neurosci..

[bb0680] Kamath Tushar, Abdulraouf Abdulraouf, Burris S.J. (2022). Single-cell genomic profiling of human dopamine neurons identifies a population that selectively degenerates in Parkinson’s disease. Nat. Neurosci..

[bb0685] Kemppainen Jukka, Aalto Sargo, Fujimoto Toshihiko (2005). High intensity exercise decreases global brain glucose uptake in humans. J. Physiol..

[bb0690] Keren-Shaul Hadas, Spinrad Amit, Weiner Assaf (2017). A unique microglia type associated with restricting development of Alzheimer’s disease. Cell.

[bb0695] Kettenmann Helmut, Hanisch Uwe-Karsten, Noda Mami, Verkhratsky Alexei (2011). Physiology of microglia. Physiol. Rev..

[bb0700] Kleele Tatjana, Rey Timo, Winter Julius (2021). Distinct fission signatures predict mitochondrial degradation or biogenesis. Nature.

[bb0705] Ko Hyoung-Gon, Chun Heejung, Han Seunghyo, Kaang Bong-Kiun (2023). Role of spinal astrocytes through the perisynaptic astrocytic process in pathological pain. Mol. Brain.

[bb0710] Koekkoek Laura L., Mul Joram D., la Fleur Susanne E. (2017). Glucose-sensing in the reward system. Front. Neurosci..

[bb0715] Koepsell Hermann (2020). Glucose transporters in brain in health and disease. Pflugers Arch. - Eur. J. Physiol..

[bb0720] Krüger R., Kuhn W., Müller T. (1998). Ala30Pro mutation in the gene encoding alpha-synuclein in Parkinson’s disease. Nat. Genet..

[bb0725] Kuhn Sarah, Gritti Laura, Crooks Daniel, Dombrowski Yvonne (2019). Oligodendrocytes in development, myelin generation and beyond. Cells.

[bb0730] Kwon Hyuk Sung, Koh Seong-Ho (2020). Neuroinflammation in neurodegenerative disorders: the roles of microglia and astrocytes. Transl. Neurodegener..

[bb0735] Langston J.W., Ballard P., Tetrud J.W., Irwin I. (1983). Chronic Parkinsonism in humans due to a product of meperidine-analog synthesis. Science (New York, N.Y.).

[bb0740] Legati A., Ghezzi D. (2023). Parkinson’s disease, parkinsonisms, and mitochondria: the role of nuclear and mitochondrial DNA. Curr. Neurol. Neurosci. Rep..

[bb0745] Leng Lige, Yuan Ziqi, Pan Ruiyuan (2022). Microglial hexokinase 2 deficiency increases ATP generation through lipid metabolism leading to β-amyloid clearance. Nat. Metab..

[bb0750] Lennie Peter (2003). The cost of cortical computation. Curr. Biol..

[bb0755] Lesage Suzanne, Brice Alexis (2012). Role of Mendelian genes in ‘sporadic’ Parkinson’s disease. Parkinsonism Relat. Disord..

[bb0760] Lewy Friedrich Heinrich (1912). Handbuch der Neurologie.

[bb0765] Li Lei, Cheng Shu-Qi, Sun Yu-Qin (2023). Resolvin D1 reprograms energy metabolism to promote microglia to phagocytize neutrophils after ischemic stroke. Cell Rep..

[bb0770] Li Xuefeng, Chen Chunhai, Pan Ting (2024). Trends and hotspots in non-motor symptoms of Parkinson’s disease: a 10-year bibliometric analysis. Front. Aging Neurosci..

[bb0775] Liang L.P., Ho Y.S., Patel M. (2000). Mitochondrial superoxide production in kainate-induced hippocampal damage. Neuroscience.

[bb0780] Liu Yi, Zhu Xiongwei (2017). Endoplasmic reticulum-mitochondria tethering in neurodegenerative diseases. Transl. Neurodegener..

[bb0785] Liu Jian, Lillo Concepción, Jonsson P. Andreas (2004). Toxicity of familial ALS-linked SOD1 mutants from selective recruitment to spinal mitochondria. Neuron.

[bb0790] Liu Lucy, MacKenzie Kevin R., Putluri Nagireddy, Maletić-Savatić Mirjana, Bellen Hugo J. (2017). The glia-neuron lactate shuttle and elevated ROS promote lipid synthesis in neurons and lipid droplet accumulation in glia via APOE/D. Cell Metab..

[bb0795] Liu Jin, Liu Yi, Gao Chao (2025). The ultrastructural and proteomic analysis of mitochondria-associated endoplasmic reticulum membrane in the midbrain of a Parkinson’s disease mouse model. Aging Cell.

[bb0800] López-Doménech Guillermo, Kittler Josef T. (2023). Mitochondrial regulation of local supply of energy in neurons. Curr. Opin. Neurobiol..

[bb0805] López-Ojeda Wilfredo, Hurley Robin A. (2023). Ketone bodies and brain metabolism: new insights and perspectives for neurological diseases. J. Neuropsychiatr. Clin. Neurosci..

[bb0810] Lorenz Carmen, Lesimple Pierre, Bukowiecki Raul (2017). Human iPSC-derived neural progenitors are an effective drug discovery model for neurological mtDNA disorders. Cell Stem Cell.

[bb0815] Mächler Philipp, Wyss Matthias T., Elsayed Maha (2016). In vivo evidence for a lactate gradient from astrocytes to neurons. Cell Metab..

[bb0820] MacVicar Brian A., Newman Eric A. (2015). Astrocyte regulation of blood flow in the brain. Cold Spring Harb. Perspect. Biol..

[bb0825] Magistretti Pierre J., Allaman Igor (2015). A cellular perspective on brain energy metabolism and functional imaging. Neuron.

[bb0830] Magistretti Pierre J., Allaman Igor (2018). Lactate in the brain: from metabolic end-product to signalling molecule. Nat. Rev. Neurosci..

[bb0835] Maker H.S., Weiss C., Silides D.J., Cohen G. (1981). Coupling of dopamine oxidation (monoamine oxidase activity) to glutathione oxidation via the generation of hydrogen peroxide in rat brain homogenates. J. Neurochem..

[bb0840] Manczak Maria, Anekonda Thimmappa S., Henson Edward, Park Byung S., Quinn Joseph, Hemachandra Reddy P. (2006). Mitochondria are a direct site of a beta accumulation in Alzheimer’s disease neurons: implications for free radical generation and oxidative damage in disease progression. Hum. Mol. Genet..

[bb0845] Mangrulkar Shubhada V., Wankhede Nitu L., Kale Mayur B. (2023). Mitochondrial dysfunction as a signaling target for therapeutic intervention in major neurodegenerative disease. Neurotox. Res..

[bb0850] Mansour Heba M., Fawzy Hala M., El-Khatib Aiman S., Khattab Mahmoud M. (2021). Inhibition of mitochondrial pyruvate carrier 1 by lapatinib ditosylate mitigates Alzheimer’s-like disease in D-galactose/ovariectomized rats. Neurochem. Int..

[bb0855] Mansour Heba M., Mohamed Ahmed F., Khattab Mahmoud M., El-Khatib Aiman S. (2023). Lapatinib ditosylate rescues motor deficits in rotenone-intoxicated rats: potential repurposing of anti-cancer drug as a disease-modifying agent in Parkinson’s disease. Eur. J. Pharmacol..

[bb0860] Marin Samantha E., Mesterman Ronit, Robinson Brian, Rodenburg Richard J., Smeitink Jan, Tarnopolsky Mark A. (2013). Leigh syndrome associated with mitochondrial complex I deficiency due to novel mutations in NDUFV1 and NDUFS2. Gene.

[bb0865] Marschallinger Julia, Iram Tal, Zardeneta Macy (2020). Lipid-droplet-accumulating microglia represent a dysfunctional and proinflammatory state in the aging brain. Nat. Neurosci..

[bb0870] Martin J.B., Gusella J.F. (1986). Huntington’s disease. Pathogenesis and management. N. Engl. J. Med..

[bb0875] Marty Nell, Dallaporta Michel, Foretz Marc (2005). Regulation of glucagon secretion by glucose transporter type 2 (Glut2) and astrocyte-dependent glucose sensors. J. Clin. Invest..

[bb0880] McHale-Owen Harriet, Faller Kiterie M.E., Chaytow Helena, Gillingwater Thomas H. (2025). Phosphoglycerate kinase 1 as a therapeutic target in neurological disease. Trends Mol. Med..

[bb0885] Medway C., Morgan K. (2014). Review: the genetics of Alzheimer’s disease; putting flesh on the bones. Neuropathol. Appl. Neurobiol..

[bb0890] Mehta Arpan R., Gregory Jenna M., Dando Owen (2021). Mitochondrial bioenergetic deficits in C9orf72 amyotrophic lateral sclerosis motor neurons cause dysfunctional axonal homeostasis. Acta Neuropathol..

[bb0895] Meles Sanne K., Teune Laura K., De Jong Bauke M., Dierckx Rudi A., Leenders Klaus L. (2017). Metabolic imaging in Parkinson disease. J. Nucl. Med..

[bb0900] Melov Simon, Adlard Paul A., Morten Karl (2007). Mitochondrial oxidative stress causes hyperphosphorylation of tau. PLoS One.

[bb0905] Mena M.A., Davila V., Sulzer D. (1997). Neurotrophic effects of L-DOPA in postnatal midbrain dopamine neuron/cortical astrocyte cocultures. J. Neurochem..

[bb0910] Merlini Mario, Meyer Eric P., Ulmann-Schuler Alexandra, Nitsch Roger M. (2011). Vascular β-amyloid and early astrocyte alterations impair cerebrovascular function and cerebral metabolism in transgenic arcAβ mice. Acta Neuropathol..

[bb0915] Meyer Dylan J., Díaz-García Carlos Manlio, Nathwani Nidhi, Rahman Mahia, Yellen Gary (2022). The Na+/K+ pump dominates control of glycolysis in hippocampal dentate granule cells. eLife.

[bb0920] Mi Yashi, Qi Guoyuan, Vitali Francesca (2023). Loss of fatty acid degradation by astrocytic mitochondria triggers neuroinflammation and neurodegeneration. Nat. Metab..

[bb0925] Mizuno Y., Ohta S., Tanaka M. (1989). Deficiencies in complex I subunits of the respiratory chain in Parkinson’s disease. Biochem. Biophys. Res. Commun..

[bb0930] Mlody Barbara, Lorenz Carmen, Inak Gizem, Prigione Alessandro (2016). Energy metabolism in neuronal/glial induction and in iPSC models of brain disorders. Semin. Cell Dev. Biol..

[bb0935] Monden Yukifumi, Mori Masato, Kuwajima Mari, Goto Tamako, Yamagata Takanori, Momoi Mariko Y. (2013). Late-onset Leigh syndrome with myoclonic epilepsy with ragged-red fibers. Brain Dev..

[bb0940] Morris Patrick J., Burke Richard D., Sharma Alok K. (2021). A comparison of the pharmacokinetics and NMDAR antagonism-associated neurotoxicity of ketamine, (2R,6R)-hydroxynorketamine and MK-801. Neurotoxicol. Teratol..

[bb0945] Mortiboys Heather, Johansen Krisztina K., Aasly Jan O., Bandmann Oliver (2010). Mitochondrial impairment in patients with Parkinson disease with the G2019S mutation in LRRK2. Neurology.

[bb0950] Mortiboys Heather, Furmston Rebecca, Bronstad Gunnar, Aasly Jan, Elliott Chris, Bandmann Oliver (2015). UDCA exerts beneficial effect on mitochondrial dysfunction in LRRK2G2019S carriers and in vivo. Neurology.

[bb0955] Mounsey Ross B., Teismann Peter (2010). Mitochondrial dysfunction in Parkinson’s disease: pathogenesis and neuroprotection. Parkinsons Dis..

[bb0960] Mukherjee Riddhita, Tompkins Carly M., Ostberg Nicolai Patrick (2022). Drp1/Fis1-dependent pathologic fission and associated damaged extracellular mitochondria contribute to macrophage dysfunction in endotoxin tolerance. Crit. Care Med..

[bb0965] Mulligan Sean J., MacVicar Brian A. (2004). Calcium transients in astrocyte endfeet cause cerebrovascular constrictions. Nature.

[bb0970] Murali Mahadevan Hariharan, Hashemiaghdam Arsalan, Ashrafi Ghazaleh, Harbauer Angelika Bettina (2021). Mitochondria in neuronal health: from energy metabolism to Parkinson’s disease. Adv. Biol..

[bb0975] Murray Kevin O., Gioscia-Ryan Rachel A., Justice Jaime N. (2026). Translational studies of chronic supplementation with a mitochondria-targeted antioxidant to improve physical function with ageing. J. Physiol..

[bb0980] Narendra Derek, Tanaka Atsushi, Suen Der-Fen, Youle Richard J. (2008). Parkin is recruited selectively to impaired mitochondria and promotes their autophagy. J. Cell Biol..

[bb0985] Narendra Derek, Tanaka Atsushi, Suen Der-Fen, Youle Richard J. (2009). Parkin-induced mitophagy in the pathogenesis of Parkinson disease. Autophagy.

[bb0990] Nguyen Thuy Trang, Ta Qui Thanh Hoai, Nguyen Thi Kim Oanh, Nguyen Thi Thuy Dung, Van Giau Vo (2020). Type 3 diabetes and its role implications in Alzheimer’s disease. Int. J. Mol. Sci..

[bb0995] Nicklas W.J., Vyas I., Heikkila R.E. (1985). Inhibition of NADH-linked oxidation in brain mitochondria by 1-methyl-4-phenyl-pyridine, a metabolite of the neurotoxin, 1-methyl-4-phenyl-1,2,5,6-tetrahydropyridine. Life Sci..

[bb1000] Niemann Axel, Ruegg Marcel, La Padula Veronica, Schenone Angelo, Suter Ueli (2005). Ganglioside-induced differentiation associated protein 1 is a regulator of the mitochondrial network. J. Cell Biol..

[bb1005] Niemann Axel, Wagner Konstanze Marion, Ruegg Marcel, Suter Ueli (2009). GDAP1 mutations differ in their effects on mitochondrial dynamics and apoptosis depending on the mode of inheritance. Neurobiol. Dis..

[bb1010] Nuytemans Karen, Theuns Jessie, Cruts Marc, Van Broeckhoven Christine (2010). Genetic etiology of Parkinson disease associated with mutations in the SNCA, PARK2, PINK1, PARK7, and LRRK2 genes: a mutation update. Hum. Mutat..

[bb1015] Omori Naomi Elyse, Woo Geoffrey Hubert, Mansor Latt Shahril (2022). Exogenous ketones and lactate as a potential therapeutic intervention for brain injury and neurodegenerative conditions. Front. Hum. Neurosci..

[bb1020] Öz Gülin, Alger Jeffry R., Barker Peter B. (2014). Clinical proton MR spectroscopy in central nervous system disorders. Radiology.

[bb1025] Padamsey Zahid, Rochefort Nathalie L. (2023). Paying the brain’s energy bill. Curr. Opin. Neurobiol..

[bb1030] Pak Kyoungjune, Malén Tuulia, Santavirta Severi (2023). Brain glucose metabolism and aging: a 5-year longitudinal study in a large positron emission tomography cohort. Diabetes Care.

[bb1035] Pang Mingli, Wang Shidi, Shi Tianyi, Chen Jinsong (2025). Overview of MitoQ on prevention and management of cardiometabolic diseases: a scoping review. Front. Cardiovasc. Med..

[bb1040] Panicker Nikhil, Kam Tae-In, Wang Hu (2022). Neuronal NLRP3 is a Parkin substrate that drives neurodegeneration in Parkinson’s disease. Neuron.

[bb1045] Panov Alexander V., Gutekunst Claire-Anne, Leavitt Blair R. (2002). Early mitochondrial calcium defects in Huntington’s disease are a direct effect of polyglutamines. Nat. Neurosci..

[bb1050] Park Jeehye, Lee Gina, Chung Jongkyeong (2009). The PINK1-Parkin pathway is involved in the regulation of mitochondrial remodeling process. Biochem. Biophys. Res. Commun..

[bb1055] Parker W.D., Boyson S.J., Parks J.K. (1989). Abnormalities of the electron transport chain in idiopathic Parkinson’s disease. Ann. Neurol..

[bib1542] Parkinson James. (1817). An essay on the shaking palsy. J. Neuropsychiatry Clin. Neurosci..

[bb1060] Patel M., Liang L.P., Roberts L.J. (2001). Enhanced hippocampal F2-isoprostane formation following kainate-induced seizures. J. Neurochem..

[bb1065] Payne Thomas, Appleby Matthew, Buckley Ellen (2023). A double-blind, randomized, placebo-controlled trial of ursodeoxycholic acid (UDCA) in Parkinson’s disease. Mov. Disord..

[bb1070] Pellerin L., Magistretti P.J. (1994). Glutamate uptake into astrocytes stimulates aerobic glycolysis: a mechanism coupling neuronal activity to glucose utilization. Proc. Natl. Acad. Sci. USA.

[bb1075] Peralta Cecilia, Biafore Federico, Depetris Tamara Soto, Bastianello Maria (2019). Recent advancement and clinical implications of 18FDG-PET in Parkinson’s disease, atypical parkinsonisms, and other movement disorders. Curr. Neurol. Neurosci. Rep..

[bib1541] Pfeiffer-Guglielmi Brigitte, Burkhard Fleckenstein, Günther Jung, Bernd Hamprecht (2003). Immunocytochemical localization of glycogen phosphorylase Isozymes in rat nervous tissues by using isozyme-specific antibodies. J. Neurochem..

[bb1080] Phelps C.H. (1972). Barbiturate-induced glycogen accumulation in brain. An electron microscopic study. Brain Res..

[bb1085] Pickett Eleanor K., Rose Jamie, McCrory Caoimhe (2018). Region-specific depletion of synaptic mitochondria in the brains of patients with Alzheimer’s disease. Acta Neuropathol..

[bb1090] Pietrocola Federico, Galluzzi Lorenzo, Pedro José Manuel Bravo-San, Madeo Frank, Kroemer Guido (2015). Acetyl coenzyme A: a central metabolite and second messenger. Cell Metab..

[bb1095] Pilic Johannes, Gottschalk Benjamin, Bourgeois Benjamin (2024). Hexokinase 1 forms rings that regulate mitochondrial fission during energy stress. Mol. Cell.

[bb1100] Pissadaki Eleftheria K., Bolam J. Paul (2013). The energy cost of action potential propagation in dopamine neurons: clues to susceptibility in Parkinson’s disease. Front. Comput. Neurosci..

[bb1105] Poewe Werner, Seppi Klaus, Tanner Caroline M. (2017). Parkinson disease. Nat. Rev. Dis. Primers.

[bb1110] Polymeropoulos M.H., Lavedan C., Leroy E. (1997). Mutation in the alpha-synuclein gene identified in families with Parkinson’s disease. Science (New York, N.Y.).

[bb1115] Poole Angela C., Thomas Ruth E., Andrews Laurie A., McBride Heidi M., Whitworth Alexander J., Pallanck Leo J. (2008). The PINK1/parkin pathway regulates mitochondrial morphology. Proc. Natl. Acad. Sci. USA.

[bb1120] Porras Omar H., Loaiza Anitsi, Felipe Barros L. (2004). Glutamate mediates acute glucose transport inhibition in hippocampal neurons. J. Neurosci..

[bb1125] Qi Xin, Qvit Nir, Su Yu-Chin, Mochly-Rosen Daria (2013). A novel Drp1 inhibitor diminishes aberrant mitochondrial fission and neurotoxicity. J. Cell Sci..

[bb1130] Rahman Shamima (2012). Mitochondrial disease and epilepsy. Dev. Med. Child Neurol..

[bb1135] Rajendran Lawrence, Paolicelli Rosa C. (2018). Microglia-mediated synapse loss in Alzheimer’s disease. J. Neurosci..

[bb1140] Rassow, Hauser, Netzker, Deutzmann (2008).

[bb1145] Reddy P. Hemachandra, Reddy Tejaswini P., Manczak Maria, Calkins Marcus J., Shirendeb Ulziibat, Mao Peizhong (2011). Dynamin-related protein 1 and mitochondrial fragmentation in neurodegenerative diseases. Brain Res. Rev..

[bb1150] Reichmann H., Janetzky B., Bischof F. (1994). Unaltered respiratory chain enzyme activity and mitochondrial DNA in skeletal muscle from patients with idiopathic Parkinson’s syndrome. Eur. Neurol..

[bb1155] Rossiello Francesca, Jurk Diana, Passos João F., d’Adda di Fagagna Fabrizio (2022). Telomere dysfunction in ageing and age-related diseases. Nat. Cell Biol..

[bb1160] Rubio Carmen, López-Landa Alejandro, Romo-Parra Hector, Rubio-Osornio Moisés (2025). Impact of the ketogenic diet on neurological diseases: a review. Life.

[bb1165] Saft Carsten, Zange Jochen, Andrich Jürgen (2005). Mitochondrial impairment in patients and asymptomatic mutation carriers of Huntington’s disease. Mov. Disord..

[bb1170] Salmon Eric, Collette Fabienne, Bastin Christine (2024). Cerebral glucose metabolism in Alzheimer’s disease. Cortex.

[bb1175] Santoro Aurelia, Balbi Valentina, Balducci Elisa (2010). Evidence for sub-haplogroup H5 of mitochondrial DNA as a risk factor for late onset Alzheimer’s disease. PLoS One.

[bb1180] Sasane Rahul, Bartels Amy, Field Michelle (2021). Parkinson disease among patients treated for benign prostatic hyperplasia with Α1 adrenergic receptor antagonists. J. Clin. Invest..

[bb1185] Sathe Abhishek G., Tuite Paul, Chen Chi (2020). Pharmacokinetics, safety, and tolerability of orally administered ursodeoxycholic acid in patients with Parkinson’s disease—a pilot study. J. Clin. Pharmacol..

[bb1190] Schapira A.H., Cooper J.M., Dexter D., Jenner P., Clark J.B., Marsden C.D. (1989). Mitochondrial complex I deficiency in Parkinson’s disease. Lancet (London, England).

[bb1195] Schmidt Saskia, Linnartz Bettina, Mendritzki Sonja (2011). Genetic mouse models for Parkinson’s disease display severe pathology in glial cell mitochondria. Hum. Mol. Genet..

[bb1200] Schousboe Arne, Bak Lasse K., Waagepetersen Helle S. (2013). Astrocytic control of biosynthesis and turnover of the neurotransmitters glutamate and GABA. Front. Endocrinol..

[bb1205] Schultz Jordan L., Brinker Alivia N., Xu Jia (2022). A pilot to assess target engagement of terazosin in Parkinson’s disease. Parkinsonism Relat. Disord..

[bb1210] Schultz Jordan L., Gander Phillip E., Workman Craig D. (2025). A dose-finding study shows terazosin enhanced energy metabolism in neurologically healthy adults. J. Parkinsons Dis..

[bb1215] Sharp F.R., Kauer J.S., Shepherd G.M. (1975). Local sites of activity-related glucose metabolism in rat olfactory bulb during olfactory stimulation. Brain Res..

[bb1220] Shaw P. (2005). Molecular and cellular pathways of neurodegeneration in motor neurone disease. J. Neurol. Neurosurg. Psychiatry.

[bb1225] Shigetomi Eiji, Kracun Sebastian, Sofroniew Michael V., Khakh Baljit S. (2010). A genetically targeted optical sensor to monitor calcium signals in astrocyte processes. Nat. Neurosci..

[bb1230] Shimura H., Hattori N., Kubo S.I. (2000). Familial Parkinson disease gene product, parkin, is a ubiquitin-protein ligase. Nat. Genet..

[bb1235] Shinn Lindsey J., Lagalwar Sarita (2021). Treating neurodegenerative disease with antioxidants: efficacy of the bioactive phenol resveratrol and mitochondrial-targeted MitoQ and SkQ. Antioxidants.

[bb1240] Shoshan-Barmatz Varda, Nahon-Crystal Edna, Shteinfer-Kuzmine Anna, Gupta Rajeev (2018). VDAC1, mitochondrial dysfunction, and Alzheimer’s disease. Pharmacol. Res..

[bb1245] Sidoryk-Węgrzynowicz Marta, Adamiak Kamil, Strużyńska Lidia (2024). Astrocyte–neuron interaction via the glutamate–glutamine cycle and its dysfunction in tau-dependent neurodegeneration. Int. J. Mol. Sci..

[bb1250] Siegmund Stephanie E., Grassucci Robert, Carter Stephen D. (2018). Three-dimensional analysis of mitochondrial crista ultrastructure in a patient with Leigh syndrome by in situ cryoelectron tomography. iScience.

[bb1255] Simmering Jacob E., Welsh Michael J., Schultz Jordan, Narayanan Nandakumar S. (2022). Use of glycolysis-enhancing drugs and risk of Parkinson’s disease. Mov. Disord..

[bb1260] Singer T.P., Salach J.I., Castagnoli N., Trevor A. (1986). Interactions of the neurotoxic amine 1-methyl-4-phenyl-1,2,3,6-tetrahydropyridine with monoamine oxidases. Biochem. J..

[bb1265] Singh Francois, Prescott Alan R., Rosewell Philippa, Ball Graeme, Reith Alastair D., Ganley Ian G. (2021). Pharmacological rescue of impaired mitophagy in Parkinson’s disease-related LRRK2 G2019S knock-in mice. eLife.

[bb1270] Sokoloff L., Takahashi S., Gotoh J., Driscoll B.F., Law M.J. (1996). Contribution of astroglia to functionally activated energy metabolism. Dev. Neurosci..

[bb1275] Somjen G.G. (1979). Extracellular potassium in the mammalian central nervous system. Annu. Rev. Physiol..

[bb1280] Staricha Kelly, Meyers Nicholas, Garvin Jodi (2020). Effect of high glucose condition on glucose metabolism in primary astrocytes. Brain Res..

[bb1285] Stoessl A. Jon, Lehericy Stephane, Strafella Antonio P. (2014). Imaging insights into basal ganglia function, Parkinson’s disease, and dystonia. Lancet (London, England).

[bb1290] Strauss Mike, Hofhaus Götz, Schröder Rasmus R., Kühlbrandt Werner (2008). Dimer ribbons of ATP synthase shape the inner mitochondrial membrane. EMBO J..

[bb1295] Stringer Danielle M., Zahradka Peter, Taylor Carla G. (2015). Glucose transporters: cellular links to hyperglycemia in insulin resistance and diabetes. Nutr. Rev..

[bb1300] Su Yu-Chin, Qi Xin (2013). Inhibition of excessive mitochondrial fission reduced aberrant autophagy and neuronal damage caused by LRRK2 G2019S mutation. Hum. Mol. Genet..

[bb1305] Sundqvist Nicolas, Sten Sebastian, Thompson Peter, Andersson Benjamin Jan, Engström Maria, Cedersund Gunnar (2022). Mechanistic model for human brain metabolism and its connection to the neurovascular coupling. PLoS Comput. Biol..

[bb1310] Surmeier D. James, Guzman Jaime N., Sanchez-Padilla Javier (2010). Calcium, cellular aging, and selective neuronal vulnerability in Parkinson’s disease. Cell Calcium.

[bb1315] Suzuki Akinobu, Stern Sarah A., Bozdagi Ozlem (2011). Astrocyte-neuron lactate transport is required for long-term memory formation. Cell.

[bb1320] Takahashi Kazutoshi, Yamanaka Shinya (2006). Induction of pluripotent stem cells from mouse embryonic and adult fibroblast cultures by defined factors. Cell.

[bb1325] Takahashi Kazutoshi, Tanabe Koji, Ohnuki Mari (2007). Induction of pluripotent stem cells from adult human fibroblasts by defined factors. Cell.

[bb1330] Tan D.X., Manchester L.C., Reiter R.J., Qi W., Kim S.J., El-Sokkary G.H. (1998). Melatonin protects hippocampal neurons in vivo against kainic acid-induced damage in mice. J. Neurosci. Res..

[bb1335] Tan Yu-Yan, Jenner Peter, Chen Sheng-Di (2022). Monoamine oxidase-B inhibitors for the treatment of Parkinson’s disease: past, present, and future. J. Parkinsons Dis..

[bb1340] Thomas Kelly Jean, McCoy Melissa K., Blackinton Jeff (2011). DJ-1 acts in parallel to the PINK1/parkin pathway to control mitochondrial function and autophagy. Hum. Mol. Genet..

[bb1345] Tretiakoff Konstantin (1919).

[bb1350] Tsogtbaatar Enkhtuul, Landin Chelsea, Minter-Dykhouse Katherine, Folmes Clifford D.L. (2020). Energy metabolism regulates stem cell pluripotency. Front. Cell Dev. Biol..

[bb1355] van der Brug Marcel P., Blackinton Jeff, Chandran Jayanth (2008). RNA binding activity of the recessive parkinsonism protein DJ-1 supports involvement in multiple cellular pathways. Proc. Natl. Acad. Sci. USA.

[bb1360] Van Der Heide Lars P., Kamal Amer, Artola Alain, Gispen Willem Hendrik, Ramakers Geert M.J. (2005). Insulin modulates hippocampal activity-dependent synaptic plasticity in a N-methyl‑d-aspartate receptor and phosphatidyl-inositol-3-kinase-dependent manner. J. Neurochem..

[bb1365] Verberne Anthony J.M., Sabetghadam Azadeh, Korim Willian S. (2014). Neural pathways that control the glucose counterregulatory response. Front. Neurosci..

[bb1370] Vijay Nisha, Morse Bridget L., Morris Marilyn E. (2015). A novel monocarboxylate transporter inhibitor as a potential treatment strategy for γ-hydroxybutyric acid overdose. Pharm. Res..

[bb1375] Vijayvergiya Chetan, Flint Beal M., Buck Jochen, Manfredi Giovanni (2005). Mutant superoxide dismutase 1 forms aggregates in the brain mitochondrial matrix of amyotrophic lateral sclerosis mice. J. Neurosci..

[bb1380] Vilchez David, Ros Susana, Cifuentes Daniel (2007). Mechanism suppressing glycogen synthesis in neurons and its demise in progressive myoclonus epilepsy. Nat. Neurosci..

[bb1385] Vlassenko Andrei G., Vaishnavi S. Neil, Couture Lars (2010). Spatial correlation between brain aerobic glycolysis and amyloid-β (Aβ) deposition. Proc. Natl. Acad. Sci. USA.

[bb1390] Von Stockum Sophia, Nardin Alice, Schrepfer Emilie, Ziviani Elena (2016). Mitochondrial dynamics and mitophagy in Parkinson’s disease: a fly point of view. Neurobiol. Dis..

[bb1395] Voorsluijs Valérie, Skupin Alexander (2025). Calcium-mediated mitochondrial energy deficiency in Parkinson’s and Alzheimer’s diseases: insights from computational modelling. Curr. Opin. Syst. Biol..

[bb1400] Walter Jonas, Bolognin Silvia, Antony Paul M.A. (2019). Neural stem cells of Parkinson’s disease patients exhibit aberrant mitochondrial morphology and functionality. Stem Cell Rep..

[bb1405] Wang Xinglong, Su Bo, Lee Hyoung-gon (2009). Impaired balance of mitochondrial fission and fusion in Alzheimer’s disease. J. Neurosci..

[bb1410] Wang Wenzhang, Wang Luwen, Lu Junjie (2016). The inhibition of TDP-43 mitochondrial localization blocks its neuronal toxicity. Nat. Med..

[bb1415] Wang Yujuan, Dai Xinyan, Li Hui (2023). The role of mitochondrial dynamics in disease. MedComm.

[bb1420] Watts Michelle E., Pocock Roger, Claudianos Charles (2018). Brain energy and oxygen metabolism: emerging role in normal function and disease. Front. Mol. Neurosci..

[bb1425] Weihofen Andreas, Thomas Kelly Jean, Ostaszewski Beth L., Cookson Mark R., Selkoe Dennis J. (2009). Pink1 forms a multiprotein complex with miro and Milton, linking Pink1 function to mitochondrial trafficking. Biochemistry.

[bb1430] Wenk Gary L. (2006). Neuropathologic changes in Alzheimer’s disease: potential targets for treatment. J. Clin. Psychiatry.

[bb1435] West A. Phillip, Khoury-Hanold William, Staron Matthew (2015). Mitochondrial DNA stress primes the antiviral innate immune response. Nature.

[bb1440] Weykopf Beatrice, Haupt Simone, Jungverdorben Johannes (2019). Induced pluripotent stem cell-based modeling of mutant LRRK2-associated Parkinson’s disease. Eur. J. Neurosci..

[bb1445] Wijesekera Lokesh C., Nigel Leigh P. (2009). Amyotrophic lateral sclerosis. Orphanet J. Rare Dis..

[bb1450] Wilson David F. (2017). Oxidative phosphorylation: regulation and role in cellular and tissue metabolism. J. Physiol..

[bb1455] Wong-Riley M.T. (1989). Cytochrome oxidase: an endogenous metabolic marker for neuronal activity. Trends Neurosci..

[bb1460] Wu Long, Zhang Xin, Zhao Liqin (2018). Human ApoE isoforms differentially modulate brain glucose and ketone body metabolism: implications for Alzheimer’s disease risk reduction and early intervention. J. Neurosci..

[bb1465] Wu Wei, Zhang Xixi, Xing Jia (2025). MitoQ alleviates prion-induced neurodegeneration by modulating DRP1- and OPA1-mediated mitochondrial dynamics. Free Radic. Biol. Med..

[bb1470] Xie Hong, Guan Jisong, Borrelli Laura A., Xu Jing, Serrano-Pozo Alberto, Bacskai Brian J. (2013). Mitochondrial alterations near amyloid plaques in an Alzheimer’s disease mouse model. J. Neurosci..

[bb1475] Xu Honghao, Chen Kefan, Zhu Haitao (2023). Neurovascular coupling changes in patients with magnetic resonance imaging negative focal epilepsy. Epilepsy Behav..

[bb1480] Xue Xiangli, Liu Beibei, Hu Jingyun, Bian Xuepeng, Lou Shujie (2022). The potential mechanisms of lactate in mediating exercise-enhanced cognitive function: a dual role as an energy supply substrate and a signaling molecule. Nutr. Metab..

[bb1485] Yan Junqiang, Sun Wenjie, Shen Mengmeng (2022). Idebenone improves motor dysfunction, learning and memory by regulating mitophagy in MPTP-treated mice. Cell Death Discov..

[bb1490] Yang Ben, Romanov Roman A., Xu Jinbin, Mothet Jean-Pierre (2023). Editorial: brain dopaminergic mechanisms. Front. Synaptic Neurosci..

[bb1495] Yin Fei, Sancheti Harsh, Patil Ishan, Cadenas Enrique (2016). Energy metabolism and inflammation in brain aging and Alzheimer’s disease. Free Radic. Biol. Med..

[bb1500] Yu Alvin, Salazar Héctor, Plested Andrew J.R., Lau Albert Y. (2018). Neurotransmitter funneling optimizes glutamate receptor kinetics. Neuron.

[bb1505] Yue M., Hinkle K.M., Davies P. (2015). Progressive dopaminergic alterations and mitochondrial abnormalities in LRRK2 G2019S knock-in mice. Neurobiol. Dis..

[bb1510] Zala Diana, Hinckelmann Maria-Victoria, Yu Hua (2013). Vesicular glycolysis provides on-board energy for fast axonal transport. Cell.

[bb1515] Zhang Y., Gao J., Chung K.K., Huang H., Dawson V.L., Dawson T.M. (2000). Parkin functions as an E2-dependent ubiquitin- protein ligase and promotes the degradation of the synaptic vesicle-associated protein, CDCrel-1. Proc. Natl. Acad. Sci. USA.

[bb1520] Zhang Yong-mei, Qi Ying-bei, Gao Ya-nan (2023). Astrocyte metabolism and signaling pathways in the CNS. Front. Neurosci..

[bb1525] Zhang Shizheng, Li Mengmeng, Li Yuan (2025). Mitochondria-targeted nanovesicles for ursodeoxycholic acid delivery to combat neurodegeneration by ameliorating mitochondrial dysfunction. J. Nanobiotechnol..

[bb1530] Zhang Yanqing, Ren Yanhong, Zhu Xiaoran (2025). Research progress on idebenone in neurodegenerative diseases. Aging Med..

[bb1535] Zimprich Alexander, Biskup Saskia, Leitner Petra (2004). Mutations in LRRK2 cause autosomal-dominant parkinsonism with pleomorphic pathology. Neuron.

[bb1540] Züchner Stephan, Mersiyanova Irina V., Muglia Maria (2004). Mutations in the mitochondrial GTPase mitofusin 2 cause Charcot-Marie-Tooth neuropathy type 2A. Nat. Genet..

